# Dual conformational recognition by Z-DNA binding protein is important for the B–Z transition process

**DOI:** 10.1093/nar/gkaa1115

**Published:** 2020-11-27

**Authors:** Chaehee Park, Xu Zheng, Chan Yang Park, Jeesoo Kim, Seul Ki Lee, Hyuk Won, Jinhyuk Choi, Yang-Gyun Kim, Hee-Jung Choi

**Affiliations:** Department of Biological Sciences, Seoul National University, Seoul 08826, Korea; Department of Chemistry, Sungkyunkwan University, Suwon 16419, Korea; Department of Chemistry, Sungkyunkwan University, Suwon 16419, Korea; Department of Biological Sciences, Seoul National University, Seoul 08826, Korea; Department of Chemistry, Sungkyunkwan University, Suwon 16419, Korea; Department of Chemistry, Sungkyunkwan University, Suwon 16419, Korea; Department of Chemistry, Sungkyunkwan University, Suwon 16419, Korea; Department of Chemistry, Sungkyunkwan University, Suwon 16419, Korea; Department of Biological Sciences, Seoul National University, Seoul 08826, Korea

## Abstract

Left-handed Z-DNA is radically different from the most common right-handed B-DNA and can be stabilized by interactions with the Zα domain, which is found in a group of proteins, such as human ADAR1 and viral E3L proteins. It is well-known that most Zα domains bind to Z-DNA in a conformation-specific manner and induce rapid B–Z transition in physiological conditions. Although many structural and biochemical studies have identified the detailed interactions between the Zα domain and Z-DNA, little is known about the molecular basis of the B–Z transition process. In this study, we successfully converted the B–Z transition-defective Zα domain, vvZα_E3L_, into a B–Z converter by improving B-DNA binding ability, suggesting that B-DNA binding is involved in the B–Z transition. In addition, we engineered the canonical B-DNA binding protein GH5 into a Zα-like protein having both Z-DNA binding and B–Z transition activities by introducing Z-DNA interacting residues. Crystal structures of these mutants of vvZα_E3L_ and GH5 complexed with Z-DNA confirmed the significance of conserved Z-DNA binding interactions. Altogether, our results provide molecular insight into how Zα domains obtain unusual conformational specificity and induce the B–Z transition.

## INTRODUCTION

Z-DNA forms biologically active left-handed double helical DNA structures (1–3). Unlike conventional right-handed B-DNA, Z-DNA has alternating *anti*- and *syn*-conformations of nucleotides, forming a unique zig-zag sugar phosphate backbone (4). Although Z-DNA is thermodynamically less stable compared to B-DNA, Z-DNA conformation could be stabilized by interactions with Z-DNA binding proteins under physiological conditions (5–10). Several Z-conformation forming segments (Z-DNA or Z-RNA) have been identified, and their physiological roles are associated with transcriptional regulation and innate immune responses (11,12). In particular, the function of Z-DNA present in a genome has been described as either transcriptional activation or repression, depending on the location and sequence-context of Z-DNA (13). Recently, it has been reported that Z-DNA binding domains (Zα domains) are involved in various cellular functions and diseases including immune responses and cancers by interacting with Z-form nucleic acids (14). For example, recognition of Z-RNA by the Zα domain of ZBP-1 was suggested to be crucial for necroptosis and inflammation (15).

A Zα domain was first identified in human ADAR1 protein (9,10,16,17). Structural studies of the Zα domain of human ADAR1 (hZα_ADAR1_) revealed that it forms a typical winged helix-turn-helix (wHTH) motif consisting of three α helices (α1, α2 and α3) and a β-wing composed of a loop between two β-strands (β2 and β3) (18,19). Thus, Zα domains belong to the family of the winged helix domain (WHD), which includes functionally diverse nucleic acid binding proteins (20,21). Not surprisingly, the overall fold of the Zα domains closely resembles that of some B-DNA binding WHDs, although there is no detectable sequence homology among them.

In general, Zα domains can both bind to the preformed Z-DNA with a high affinity as a Z-DNA binder and shift the B–Z equilibrium of a potential Z-forming DNA toward Z conformation (referred to as B–Z transition) as a B–Z converter (22,23). However, some Zα domains show different functional characteristics. For example, hZβ_ADAR1_, the second Zα domain present in human ADAR1 protein, does not have conformation-specific Z-DNA binding due to the lack of the conserved tyrosine residue located in an α3 helix (24). Another Zα domain, vvZα_E3L_, present in vaccinia viral E3L protein specifically interacts with the Z-conformation of nucleic acid duplexes, but it completely lacks a B–Z transition activity under physiological conditions (25–29). The NMR structure of the DNA-free form of vvZα_E3L_ confirmed that it possesses a common fold of the wHTH motif similar to hZα_ADAR1_ (30). However, the key tyrosine residue of vvZα_E3L_ was shown to adopt different rotamers, which would reduce Z-DNA binding and result in a lack of B–Z transition activity (26,30). This functional separation of Z-conformation binding activity and B–Z transition activity is unique in vvZα_E3L_ and raises an interesting question about the molecular mechanism of the B–Z transition.

To date, many structures of several Zα domains complexed with Z-DNA have been determined, greatly improving our understanding of the Z conformation specificity of the Zα domains (18,25,31–38). However, structural information about these detailed interactions is not enough to understand the molecular mechanism of how the B–Z transition proceeds by the Zα domain. A single-molecule study suggested a conformation selection process in which Zα specifically binds to the Z-DNA that is already formed (39). On the other hand, NMR studies and single molecule FRET experiments of hZα_ADAR1_ suggested an active B–Z transition mechanism, in which hZα_ADAR1_ first binds to the B-DNA region and then converts it to Z-DNA (40,41). In this context, hZα_ADAR1_ has been proposed to have a very interesting conformational specificity that binds to both B- and Z-DNA.

In this study, we employed the rational protein engineering strategy to identify what structural factors of the Zα domain are required for B–Z transition activity and which structural features of the Zα domain result in distinguished DNA conformation specificity compared to other nucleic acid binding proteins. First, we separately evaluated Z-DNA binding ability and protein-induced B–Z transition activity using vvZα_E3L_. Biochemical analyses of engineered vvZα_E3L_ mutants demonstrate that B-DNA binding ability is important for the B–Z transition activity. Second, using GH5, a canonical B-DNA binding protein with a wHTH motif, as a starting template, we successfully engineered it into a Z-DNA binder and B-to-Z converter by introducing a few point mutations while maintaining the structural integrity of GH5. Functional analyses of GH5 mutants show that key Z-DNA contacting residues on the α3 helix and the β-wing conformation are required for the transformation of a B-DNA binder to a Zα-like protein. To confirm that our engineered vvZα_E3L_ and GH5 proteins form correctly folded structures and interact with Z-DNA in a manner similar to hZα_ADAR1_, we determined the crystal structures of the mutants of vvZα_E3L_ and GH5 complexed with Z-DNA, respectively.

## MATERIALS AND METHODS

### Protein preparation

The Zα domain of human ADAR1 (hZα_ADAR1_: amino acids 133–199) was cloned into the pET28a vector. *Escherichia coli* Rosetta (DE3) cells transformed with this construct were grown in LB broth containing 50 μg/ml kanamycin at 37°C until the OD_600_ was around 0.5–0.6 and protein expression was induced by 0.5 mM IPTG. After an additional 4 h incubation at 30°C, cells were harvested and lysed in PBST buffer (1 × phosphate-based saline, 0.05% Tween 20 and 1 mM PMSF) supplemented with DNase I (Roche) using EmulsiFlex-C3 (AVESTIN). Cell lysates were centrifuged at 14,000 rpm for 20 min, and the supernatant was loaded onto Ni-NTA agarose resin (Qiagen) pre-equilibrated with PBST buffer. The resin was washed with 40 mM Imidazole buffer (20 mM Tris–HCl, pH 8.0, 150 mM NaCl, 40 mM Imidazole), and bound protein was eluted from the resin with 250 mM imidazole buffer (pH 8.0). The N-terminal His_6_ tag was cleaved by thrombin (Sigma-Aldrich) treatment at 4°C for 16 h in cleavage buffer (20 mM Tris–HCl, pH 8.0, 100 mM NaCl, 2.5 mM CaCl_2_, 1 mM EDTA), and cleaved protein was further purified with a HiTrap SP column (GE Healthcare) and HiLoad 16/600 Superdex 200 column (GE Healthcare) pre-equilibrated with 20 mM HEPES, pH 7.5 and 150 mM NaCl. Purified protein was dialyzed against DNA binding (DB) buffer (5 mM HEPES, pH 7.5, 20 mM NaCl) at 4°C for 16 h before storage at –80°C.

Purification of vvZα_E3L_ (aa 2–78 of *Vaccinia virus* protein E3L) and its mutants (Supplementary Table S1) followed the same procedure described above except for the use of a HiTrap Q column (GE Healthcare) instead of a HiTrap SP column.

GH5 (aa 25–100 of the globular domain of *Gallus gallus* histone H5), GH5* and its mutants (Supplementary Table S1) were purified by the same method as described above except for using 20 mM HEPES, pH 8.0 and 150 mM NaCl as gel filtration buffer. The final dialysis into DB buffer was not performed due to protein aggregation at low salt condition.

### Circular dichroism measurements

B–Z transition of DNA duplex was monitored based on the CD spectrum using a J-810 CD spectrometer (Jasco). The oligonucleotide of *d*(CG)_6_ purchased from IDT was dissolved in CD1 buffer (5 mM HEPES, pH 7.5, 0.1 mM EDTA and 10 mM NaCl) and annealed prior to use. The CD measurements of DNA duplex in the presence of vvZα_E3L_ and its mutant proteins were carried out at 25°C. Specifically, 20 μM of the DNA duplex substrate in a CD1 buffer was used for the CD measurement with a 0.1-cm quartz cell. To prevent a substantial change in buffer composition due to the addition of protein sample, the maximum volume of protein did not exceed 5% of the total volume. Before each measurement, samples were incubated for 1 h at 25°C. CD spectra between 230 and 320 nm were recorded three times and averaged. The CD measurements of GH5* and its mutant proteins were carried out in different conditions to prevent aggregation of protein/DNA complex. CD spectra were collected using a 1-cm quartz cell, and 1 μM of the DNA duplex substrate was dissolved in CD2 buffer (5 mM HEPES, pH 8.0, 50 mM NaCl and 0.1 mM EDTA). All other details for CD measurements are the same as in the CD experiment of vvZα_E3L_. The relative transition activity of each protein was calculated by comparison of each CD signal at 255 nm with that of hZα_ADAR1_ (set to 100%) during the B–Z transition. When analyzing changes in the CD signals at 255 nm, the CD signals by the protein samples were ignored because they were negligibly small (Supplementary Figure S1) (22). The relative amount of proteins to nucleic acids used for CD measurement was indicated as [P]/[N] ratio, which is the molar ratio of protein monomer to double-stranded DNA of *d*(CG)_6_. This definition was used throughout this study.

### Double-stranded DNA preparation

DNA oligonucleotides for crystallization, *d*(TCGCGCG); for CD measurements, *d*(CG)_6_; and for MST measurements, 5′-Alexa647-labeled *d*(TATGCAATCGTAATAAACCGT), and its non-labeled complementary DNA were purchased from IDT. 5′-Cy5-labeled *d*[T(Br^5^CG)_15_] was purchased from Gene Link. DNA samples were dissolved in DB buffer (for crystallization and MST) or CD1 buffer (for CD). Dissolved DNAs were fully denatured at 95°C for 5 min and were annealed by gradual cooling to 4°C to make double-stranded DNA. DNA duplex samples at appropriate concentrations (1.2 mM for crystallization, 50 μM for MST and 1 mM for CD) were stored at –20°C or used immediately.

### Crystallization

Purified vvZα_E3L_:α3_ADAR1_ (0.6 mM) was mixed with [*d*(TCGCGCG)]_2_ at a 2:1 molar ratio and incubated at room temperature for 1 h before crystallization. Crystals were obtained by the hanging drop vapor diffusion method at 22°C using 0.1 M sodium citrate (pH 4.0), 0.8 M ammonium sulfate and 10% ethylene glycol as a reservoir solution.

Similarly, purified GH5*:α3N_ADAR1_-PW (0.3 mM) was mixed with [*d*(TCGCGCG)]_2_ at a 2:1 molar ratio and incubated at room temperature for 1 h before crystallization. Crystals were obtained by the hanging drop vapor diffusion method at 22°C using 0.1 M MES (pH 6.0), 20% PEG 4000 and 5% ethylene glycol as a reservoir solution.

### Data collection and structure determination

Crystals of vvZα_E3L_:α3_ADAR1_/[*d*(TCGCGCG)]_2_ complex and GH5*:α3N_ADAR1_-PW/[*d*(TCGCGCG)]_2_ complex were frozen using 20–25% ethylene glycol as a cryoprotectant. Diffraction data sets were collected on beamline 5C at the Pohang Accelerator Laboratory (PAL, Korea). Images were processed using HKL2000 (42) and the CCP4 suite (43). The complex structures of vvZα_E3L_:α3_ADAR1_/[*d*(TCGCGCG)]_2_ and GH5*:α3N_ADAR1_-PW/[*d*(TCGCGCG)]_2_ were solved by molecular replacement with the PHENIX program (44) using the structure of the Zα domain of ADAR1 (PDB ID 1QBJ) and globular domain of histone H5 (PDB ID 1HST) as a search model, respectively. Several cycles of manual model rebuilding with COOT (45) and refinement with PHENIX (44) were performed, and each final model was validated by MolProbity (46). The final model of the vvZα_E3L_:α3_ADAR1_/[*d*(TCGCGCG)]_2_ complex consisting of three copies of vvZα_E3L_:α3_ADAR1_ and three copies of *d*(TCGCGCG) was deposited in the PDB with PDB ID 7C0I, and the model of GH5*:α3N_ADAR1_-PW/[*d*(TCGCGCG)]_2_ complex containing 2 copies of GH5*:α3N_ADAR1_-PW and 1 copy of [*d*(TCGCGCG)]_2_ was deposited in the PDB with PDB ID 7C0J. Data collection and refinement statistics are shown in Supplementary Tables S2 and S3.

### Affinity measurement by MicroScale Thermophoresis (MST)

Affinity measurements by MST (47) were performed using a Monolith NT.115 pico instrument (NanoTemper). Each labeled dsDNA sample, *d*(5′-Alexa647-TATGCAATCGTAATAAACCGT)::*d*(ACGGTTTATTACGATTGCATA) (named B-DNA) (39) and [*d*(5′-Cy5-(Br^5^CG)_15_]_2_ (named Z-DNA), was used at a concentration of 5 nM. *d*(CG) repeat DNA duplexes containing Br^5^C bases are known to form stable Z-conformations in physiological conditions (48). The prepared protein was titrated in 1:1 serial dilution in DB buffer. In the case of GH5*:α3N_ADAR1_-PW, the DB buffer was substituted by a high-salt buffer consisting of 20 mM HEPES (pH 8.0) and 100 mM NaCl or a high-salt KCl buffer consisting of 20 mM Tris–HCl (pH 7.8) and 150 mM KCl. All buffers used in the experiments were supplemented with 0.5 mg/ml BSA and 0.05% (v/v) Tween 20. After 15 min (vvZα_E3L_ and its mutants) or 10 min (GH5* and its mutants) incubation at room temperature in the dark, each protein/DNA mixture was added into a Monolith NT.115 standard capillary. The measurements were performed at 5% LED power and 40% MST power (vvZα_E3L_:α3_ADAR1_) or 80% MST power (GH5*:α3N_ADAR1_-PW) at 22°C. Data analysis by nonlinear regression was performed using MO.Affinity Analysis provided by NanoTemper and GraphPad Prism (GraphPad Software, USA).

### Multi-angle light scattering coupled with size exclusion chromatography (SEC-MALS)

MALS experiments were performed with miniDAWN TREOS (Wyatt Technology, Co.) to determine the absolute molecular mass. Each protein sample was loaded into a Superdex 200 Increase 10/300 GL column (GE healthcare) pre-equilibrated with 20 mM HEPES (pH 7.5) and 100 mM NaCl. The light scattering signal and UV absorbance at 280 nm were measured, and data were analyzed using Astra 6 software (Wyatt Technology, Co.) to assess molar mass.

## RESULTS

### B–Z transition activity of α3 helix-swapped mutants of vvZα_E3L_

**Figure 1.**
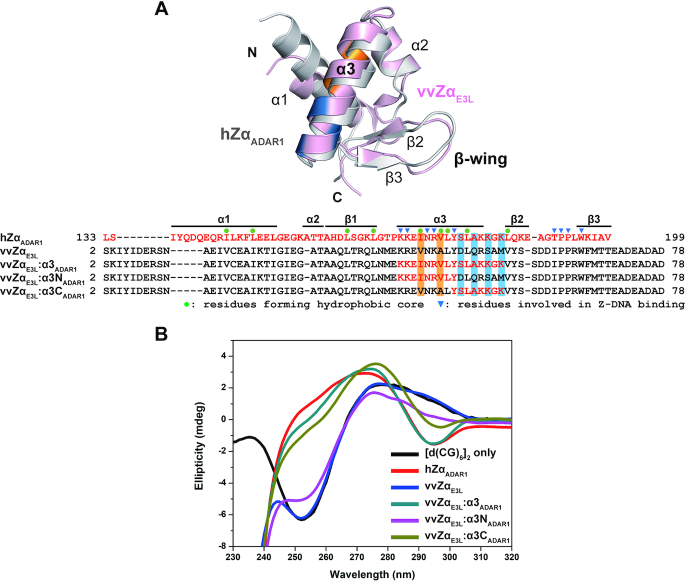
Design of α3-swapped vvZα_E3L_ mutants and their CD spectra. (**A**) Structural and sequence comparison of hZα_ADAR1_ and wild-type and chimeric mutants of vvZα_E3L_. Structural alignment of free vvZα_E3L_ (PDB ID 1OYI, light pink) and hZα_ADAR1_ (PDB ID 1QBJ, gray) and sequence alignment of hZα_ADAR1_, vvZα_E3L_ and α3-swapped vvZα_E3L_ mutants (vvZα_E3L_:α3_ADAR1_, vvZα_E3L_:α3N_ADAR1_, and vvZα_E3L_:α3C_ADAR1_) are shown. In chimeric mutants, amino acids derived from hZα_ADAR1_ are in red. Residues involved in hydrophobic core formation in hZα_ADAR1_, but are not conserved in vvZα_E3L_ are highlighted in orange. The residues highlighted in blue are the C-terminal residues of the α3 helix that are not conserved between hZα_ADAR1_ and vvZα_E3L_. These residues are represented in the ribbon diagram with the same color code. (**B**) CD spectra of *d*(CG)_6_ in the presence of α3-swapped vvZα_E3L_ mutants. The transition of *d*(CG)_6_ from B-conformation to Z-conformation was monitored in the presence of various proteins at a [P]/[N] ratio of 4 using CD. The B–Z transition of *d*(CG)_6_ induced by vvZα_E3L_ mutants are compared with that by hZα_ADAR1_.

We first confirmed that vvZα_E3L_ lacks B–Z transition activity under physiological conditions even with an excess molar ratio of [P]/[N] (Supplementary Figure S1). To investigate structural elements responsible for B–Z transition activity that are present in hZα_ADAR1_ and absent in vvZα_E3L_, we designed several chimeric mutants of vvZα_E3L_ and hZα_ADAR1_ and monitored their B–Z transition activities by CD spectroscopy. Based on structural information of hZα_ADAR1_ in complex with Z-DNA, we reasonably assumed that the regions of the α3 helix would be crucial for the B–Z transition (18,25,31–38). Thus, we initially created an α3-helix-swapped mutant of vvZα_E3L_ (vvZα_E3L_:α3_ADAR1_), in which the α3 helix of vvZα_E3L_ was replaced by the corresponding helix of hZα_ADAR1_ (Figure 1A, Supplementary Table S1, and Supplementary Figure S2). Whereas the wild-type vvZα_E3L_ has no detectable B–Z transition activity, the B–Z transition activity of vvZα_E3L_:α3_ADAR1_ was observed by CD spectroscopy using [*d*(CG)_6_]_2_ at a molar ratio of [P]/[N] = 4, i.e. four protein molecules for one double-stranded DNA molecule, which was previously reported as the stoichiometry between the Zα domain and Z-DNA (18,22,35). The B–Z transition activity of this chimera was similar to that of hZα_ADAR1_ when CD signals at 255 nm were compared (Figure 1B and Table 1). The CD titration profile of this mutant indicated that the B–Z transition reached saturation at a [P]/[N] ratio of 4 (Supplementary Figure S1). To further study the role of the α3 helix, two chimeric mutants containing either the C-terminal part (vvZα_E3L_:α3C_ADAR1_) or the N-terminal part (vvZα_E3L_:α3N_ADAR1_) of the α3 helix of hZα_ADAR1_ were constructed (Figure 1A), and we examined their B–Z transition activities. The CD spectrum by vvZα_E3L_:α3C_ADAR1_ showed a high degree of B–Z transition, almost identical to that of vvZα_E3L_:α3_ADAR1_ (Figure 1B). On the other hand, vvZα_E3L_:α3N_ADAR1_ induced a relatively small conformational change from B-DNA to Z-DNA (Figure 1B), classifying this chimera as a weak B–Z converter. Moreover, vvZα_E3L_:α3N_ADAR1_ requires excess [P]/[N] ratio to reach saturation in CD titration experiment. (Supplementary Figure S1). Altogether, we concluded that the C-terminal part of the α3 helix of hZα_ADAR1_ (α3C_ADAR1_) is more important for B–Z transition activity than is the α3N_ADAR1_.

**Table 1.** Relative B–Z transition activity of each vvZα_E3L_ mutant compared to that of hZα_ADAR1_

Protein	B–Z conversion (%)
hZα_ADAR1_	100±4.0^a^
vvZα_E3L_	0±0.1
vvZα_E3L_:α3_ADAR1_	78±3.1
vvZα_E3L_:α3N_ADAR1_	17±0.8 (61±2.3^b^)
vvZα_E3L_:α3C_ADAR1_	71±2.8
vvZα_E3L_-D49S	46±1.9 (70±2.7^b^)
vvZα_E3L_-D49R	35±1.5 (71±2.8^b^)
vvZα_E3L_-S53K	16±0.8 (65±2.5^b^)
vvZα_E3L_-M55K	7±0.3 (57±2.2^b^)
vvZα_E3L_-S53K/M55K	33±1.4 (72±2.9^b^)
vvZα_E3L_-S49S/S53K/M55K	67±2.6
vvZα_E3L_-D49R/S53K/M55K	71±2.9
vvZα_E3L_-V43I	2±0.2 (53±2.1^b^)
vvZα_E3L_-V43I/A46V	4±0.3 (57±2.3^b^)
vvZα_E3L_-A46V/53K	39±1.6 (71±2.8^b^)
vvZα_E3L_-A46V/S53K/M55K	69±2.7

^a^The relative B–Z transition activity compared to that of hZα_ADAR1_ at a [P]/[N] ratio of 4.

^b^The number in parentheses indicates the B–Z transition activity at a [P]/[N] ratio of 30.

### Effect of charge distribution in the C-terminal part of the α3 helix on B–Z transition activity

**Figure 2.**
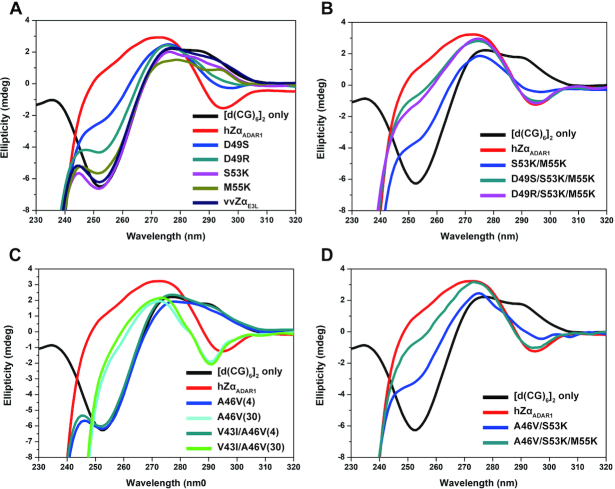
CD analysis for the B–Z transition of [*d*(CG)_6_]_2_ by vvZα_E3L_ mutants. CD spectra of *d*(CG)_6_ measured in the presence of (**A**) single-point mutation and (**B**) double- and triple-point mutations of vvZα_E3L_, which have reduced negative charge or increased positive charges are presented. Changes in the CD signals at 255 and 292 nm represent the B–Z transition of [*d*(CG)_6_]_2_. CD spectra of [*d*(CG)_6_]_2_ alone (B-DNA, Supplementary Figure S1) and in the presence of hZα_ADAR1_ or vvZα_E3L_ at a [P]/[N] ratio of 4 are shown as controls. (**C**) CD spectra of *d*(CG)_6_ measured in the presence of the hydrophobic core mutants of vvZα_E3L_ at [P]/[N] ratios of 4 and 30 are shown. The numbers 4 and 30 in parentheses represent the [P]/[N] ratios. (**D**) CD spectra of *d*(CG)_6_ measured in the presence of the combination-mutants of vvZα_E3L_ containing both positively charged and hydrophobic core mutations at a [P]/[N] ratio of 4 are shown.

To identify which amino acids in α3 helix-swapped mutants of vvZα_E3L_ are critical for B–Z transition activity, we generated point mutations in the α3 helix of vvZα_E3L_. Although the last C-terminal turn of the α3 helix (S178–K182 in hZα_ADAR1_) had no direct contact with Z-DNA based on the published structures of Zα/Z-DNA complexes, we postulated that positively charged residues of the α3C_ADAR1_ may play an important role in B–Z transition based on the CD results of vvZα_E3L_:α3C_ADAR1_. To test our hypothesis, several vvZα_E3L_ mutants containing individual or combined point mutations of D49S, D49R, S53K and M55K, which correspond to S178, K182 and K184 of hZα_ADAR1_, respectively, were generated (Supplementary Table S1 and Supplementary Figure S2). These vvZα_E3L_ mutants with a single point mutation (vvZα_E3L_-D49S, -D49R, -S53K, and -M55K) generally exhibited weak B–Z transition activities at a 4 [P]/[N] molar ratio as assessed by CD (Figure 2A and Table 1). In contrast, a double-point mutant, vvZα_E3L_-S53K/M55K, showed enhanced B–Z transition activity (Figure 2B and Table 1). Finally, the triple-point mutants (vvZα_E3L_-D49R/S53K/M55K and vvZα_E3L_-D49S/S53K/M55K) were able to convert B-DNA to Z-DNA at a 4 [P]/[N] molar ratio in a manner similar to those of vvZα_E3L_:α3_ADAR1_ and vvZα_E3L_:α3C_ADAR1_ (Figure 2B). These results indicate that removal of negatively charged residue and the addition of positively charged residues (D49R, S53K and M55K) in the α3C region of vvZα_E3L_ greatly enhance B–Z transition activity.

### Effect of hydrophobic residues in the N-terminal part of the α3 helix on B–Z transition activity

The structure of hZα_ADAR1_ revealed that nonpolar residues in α1 (I143 and L147), α2 (L161 and L165) and α3 (I172, V175, L176, and L179) helices and L185 of the β2 strand formed a hydrophobic core, which may be important for maintaining structural integrity (19). The sequence comparison of vvZα_E3L_ with hZα_ADAR1_ showed that hydrophobic core-forming Leu residues of hZα_ADAR1_ are mostly conserved in vvZα_E3L_ (L32, L36, L47 and L50), but two amino acids in the α3N region of vvZα_E3L_ has smaller aliphatic residues (V43 and A46) than hZα_ADAR1_ (I172 and V175) (Figure 1). To examine the importance of the hydrophobic residues in the α3N for B–Z transition activity, vvZα_E3L_ mutants with different hydrophobicity at α3N were created (V43I and A46V) (Supplementary Table S1 and Supplementary Figure S2). Single (vvZα_E3L_-V43I) or double point mutation (vvZα_E3L_-V43I/A46V) did not improve the B–Z transition activity at a 4 [P]/[N] molar ratio (Figure 2C). However, at a 30 [P]/[N] molar ratio, vvZα_E3L_-V43I and vvZα_E3L_-V43I/A46V showed substantial B–Z transition activities, although the transition was not complete (Figure 2C). These results suggested that a more stable hydrophobic core formed by the bigger hydrophobic residues in the α3N contributes to the B–Z transition process.

Next, we generated combination mutants of vvZα_E3L_ having hydrophobic residues in the α3N and positively charged residues in the α3C. Two mutants, vvZα_E3L_-A46V/S53K and vvZα_E3L_-A46V/S53K/M55K, showed drastically enhanced B–Z transition compared with single point mutants at a 4 [P]/[N] molar ratio (Figure 2D and Table 1). This result suggests that the N-terminal hydrophobic residues and the C-terminal positively charged residues of the α3 helix improved the B–Z transition activity in a synergistic manner. Thus, we identified key amino acid residues of the Zα domain that affect the B–Z transition.

### B-DNA binding affinity of the α3 helix-swapped vvZα_E3L_ mutants

In the previous sections, we showed that the introduction of positive charges into the α3C region of vvZα_E3L_ enhanced B–Z transition activity. To test whether increased B–Z transition activity is related to the DNA binding affinity, we determined the DNA binding affinity of each α3 chimeric vvZα_E3L_ mutant (vvZα_E3L_:α3_ADAR1_, vvZα_E3L_:α3N_ADAR1_ and vvZα_E3L_:α3C_ADAR1_) using MST (47). As summarized in Table 2, vvZα_E3L_:α3_ADAR1_ and vvZα_E3L_:α3C_ADAR1_ have significantly enhanced B-DNA binding affinities, with *K*_D_ values of 2.6 μM and 1.1 μM, respectively. Interestingly, these *K*_D_ values are almost the same as that of hZα_ADAR1_ for the interaction with B-DNA (*K*_D_ of 1.8 μM). In contrast, the wild-type vvZα_E3L_ had a much lower affinity to B-DNA (*K*_D_ of 20 μM) (Table 2 and Supplementary Figure S3). The chimeric mutant, vvZα_E3L_:α3N_ADAR1_, which did not exhibit an observable B–Z transition activity at a 4 [P]/[N] molar ratio, showed little improvement in B-DNA binding affinity with a *K*_D_ of 17 μM. Therefore, the results of our affinity measurements in combination with CD data suggest that improved B-DNA binding affinities of vvZα_E3L_ mutants (vvZα_E3L_:α3_ADAR1_ and vvZα_E3L_:α3C_ADAR1_) are closely related to the increment of B–Z transition activity. Although these two mutants showed improvement in Z-DNA binding by 2–3 times compared to vvZα_E3L_, they still have 10-fold lower affinities for Z-DNA than hZα_ADAR1_ (Table 2). Thus, high affinity binding to Z-DNA may not be necessary for B–Z transition activity. Based on our observations, we postulated that positively charged residues in the vvZα_E3L_:α3_ADAR1_ mutant may be involved in increased B-DNA binding affinity and thus may promote the B–Z transition process.

**Table 2.** DNA binding affinities of hZα_ADAR1_ and wild-type and chimeric mutants of vvZα_E3L_

Protein	Z-DNA binding affinity (μM)	B-DNA binding affinity (μM)
hZα_ADAR1_	0.018 ± 0.001	2.7 ± 0.5
vvZα_E3L_	2.0 ± 0.1	21 ± 0.5
vvZα_E3L_:α3_ADAR1_	1.0 ± 0.05	2.4 ± 0.2
vvZα_E3L_:α3N_ADAR1_	0.94 ± 0.01	18 ± 1.0
vvZα_E3L_:α3C_ADAR1_	0.74 ± 0.01	1.3 ± 0.2

All experiments were performed at 22°C with buffer consisting of 5 mM HEPES (pH 7.5), 20 mM NaCl, 0.5 mg/ml BSA and 0.05% Tween20.

### Crystal structure of engineered vvE3L in complex with [*d*(TCGCGCG)]_2_

**Figure 3.**
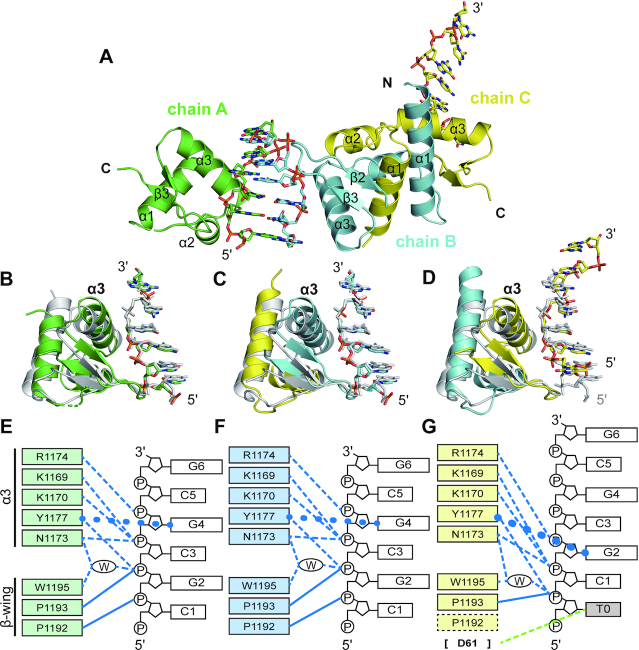
Crystal structure of vvZα_E3L_ mutant in complex with Z-DNA (vvZα_E3L_:α3_ADAR1_/Z-DNA). (**A**) Overall structure of vvZα_E3L_:α3_ADAR1_ complexed with [*d*(TCGCGCG)]_2_. Monomeric (chain A in green) and domain-swapped dimeric (chains B and C in cyan and yellow, respectively) vvZα_E3L_ mutant and Z-DNA of *d*(TCGCGCG) (chains D, E, and F) in an asymmetric unit of the crystal lattice are shown. (**B–D**) The complex structure of each DNA chain and its binding partner protein (chain A or composite models) is aligned to the complex structure of hZα_ADAR1_ and *d*(TCGCGCG) (PDB ID 1QBJ). One composite model consists of chain B (aa 37–69) and chain C (aa 6–36), and the other consists of chain B (aa 3–36) and chain C (aa 37–70). (**E–G**) Protein–DNA interactions are shown as schematic diagrams. Hydrogen bonds are shown as dashed lines, van der Waals contacts are shown as solid lines, and the CH–π interactions are shown as circled lines. Water molecule within the protein–DNA interface is marked with a W inside the oval. In the chimeric mutant of vvZα_E3L_, amino acids derived from hZα_ADAR1_ are indicated by adding 1,000 to the amino acid numbers of hZα_ADAR1_ to clarify that those residues are from foreign protein. Residues involved in Z-DNA binding are indicated by colored boxes and the residue, which does not participate in the interaction with Z-DNA is shown by dotted box. Non-canonical interaction of D61 (shown in brackets), which is not conserved between hZα_ADAR1_ and vvZα_E3L_ is indicated by a green dashed line.

To understand the functional acquisition of B–Z transition activity of chimeric vvZα_E3L_ at the molecular level, we determined the crystal structure of vvZα_E3L_:α3_ADAR1_ in complex with [*d*(TCGCGCG)]_2_ at 2.4 Å resolution (Supplementary Table S2). Unexpectedly, a monomeric form of vvZα_E3L_ chimera (chain A) and a domain-swapped dimer (chains B and C) were present with three left-handed *d*(TCGCGCG) molecules in an asymmetric unit of the crystal lattice (Figure 3A). In solution, vvZα_E3L_:α3_ADAR1_ showed increased absolute molar mass in a concentration dependent manner as analyzed by SEC-MALS, suggesting that vvZα_E3L_:α3_ADAR1_ is likely to dimerize at high concentrations (Supplementary Figure S4). A composite model consisting of the N-terminal part of chain B (3–36) and the C-terminal part of chain C (37–70) or vice versa was well aligned to the monomeric form of chimera (chain A) with an RMSD of 0.47 Å (Supplementary Figure S5).

By analyzing three molecules of the chimera/Z-DNA complex in an asymmetric unit, we discovered that the Z-DNA binding interface in one of three complex molecules (Chains C and F) was shifted by two bases, interacting with T0 as well as P1 and P2 (Figure 3D and G). Specifically, Y1177 of vvZα_E3L_ chimera (corresponding to Y48 of vvZα_E3L_) makes close contact with G2 instead of G4, and the N1173 of the vvZα_E3L_ chimera (corresponding to N44 of vvZα_E3L_) interacts with P2 instead of P4. (The residues derived from hZα_ADAR1_ in the vvZα_E3L_ chimera are numbered by adding 1,000 to the original residue numbers of hZα_ADAR1_). Although this unusual complex may be formed by crystallographic packing, it is interesting to note that the binding interface between chains C and F looks similar to that of the complex found in the B–Z junction (Supplementary Figure S6). In the remaining two complexes, a conventional Z-DNA binding interface was formed by K1169, N1173, Y1177, W1195 of the vvZα_E3L_ chimera and P3, P4, and G4 of Z-DNA (Figure 3B, C, E, and F).

Three molecules of *d*(TCGCGCG) in our complex structure are well aligned to each other with RMSD values in the range of 0.3–0.4 Å. When comparing vvZα_E3L_ chimera-bound Z-DNA with hZα_ADAR1_-bound Z-DNA, protein-free Z-DNA (PDB ID 4FS6), and ideal B-DNA generated by the Coot program (45), it was clear that the DNA in complex with vvZα_E3L_:α3_ADAR1_ formed a Z-conformation similar to a Zα-bound or a protein-free Z-DNA structure, with RMSD values of 0.6 Å and 0.8 Å, respectively (Supplementary Table S4). Analysis of the vvZα_E3L_ chimera-bound Z-DNA structure using the web 3DNA 2.0 program (49), which provides various parameters related to DNA structure including ‘Rise’ and ‘Twist,’ showed that the ‘Rise’ values are between those in the protein-free state and in the hZα_ADAR1_-bound state (Supplementary Figure S7).

When a monomeric form of vvZα_E3L_:α3_ADAR1_ (chain A) was aligned to hZα_ADAR1_ in a complex with *d*(TCGCGCG), the RMSD value was calculated as 0.52 Å. In contrast, an RMSD value of 1.87 Å was obtained by aligning vvZα_E3L_:α3_ADAR1_ with vvZα_E3L_ in the DNA-free state. This comparison clearly shows that vvZα_E3L_:α3_ADAR1_ is structurally more similar to hZα_ADAR1_; the sequence identity between vvZα_E3L_:α3_ADAR1_ and hZα_ADAR1_ is only 43% compared with 75% between vvZα_E3L_:α3_ADAR1_ and vvZα_E3L_ (Figure 1A). The large structural deviation between vvZα_E3L_:α3_ADAR1_ and DNA-free vvZα_E3L_ comes from the difference in relative position between α1 and α3 helices and the positional shift of the β-wing. When α3 helices were aligned together, the α1 helix was located much closer to the α3 helix in the vvZα_E3L_ chimera, forming a tightly packed hydrophobic core (Supplementary Figure S8). V43I and A46V mutations of the α3 helix in vvZα_E3L_ chimera contributed to the formation of the extensive hydrophobic interaction network, re-locating the α2 helix and bringing the α1 helix closer to the α3 helix. As a result, A12, V15 and M38, which do not make contact with V47 and A50 in the vvZα_E3L_ structure, interact with the corresponding residues, I1172 and V1175, of the vvZα_E3L_ chimera (Supplementary Figure S9).

Altogether, our structure shows that the engineered vvZα_E3L_ chimera, vvZα_E3L_:α3_ADAR1_, forms a Zα-like structure, which may be related to the functional acquisition of B–Z conversion activity of this chimera.

### Construction of a chimeric protein of GH5 that enables B–Z transition

It is noteworthy that some B-DNA binding proteins containing a wHTH motif show high structural similarity to hZα_ADAR1_ (18,20). In the previous sections, chimeric mutants of vvZα_E3L_ having the enhanced B-DNA binding affinity showed B–Z transition activity. Thus, we hypothesized that it would be possible to engineer B-DNA binding proteins with high structural homology to Zα domain into Zα-like proteins with B–Z transition activity by adding Z-DNA binding ability. To demonstrate our hypothesis, we created a new Zα-like protein by introducing a number of mutations into the B-DNA binding protein with a wHTH motif.

**Figure 4.**
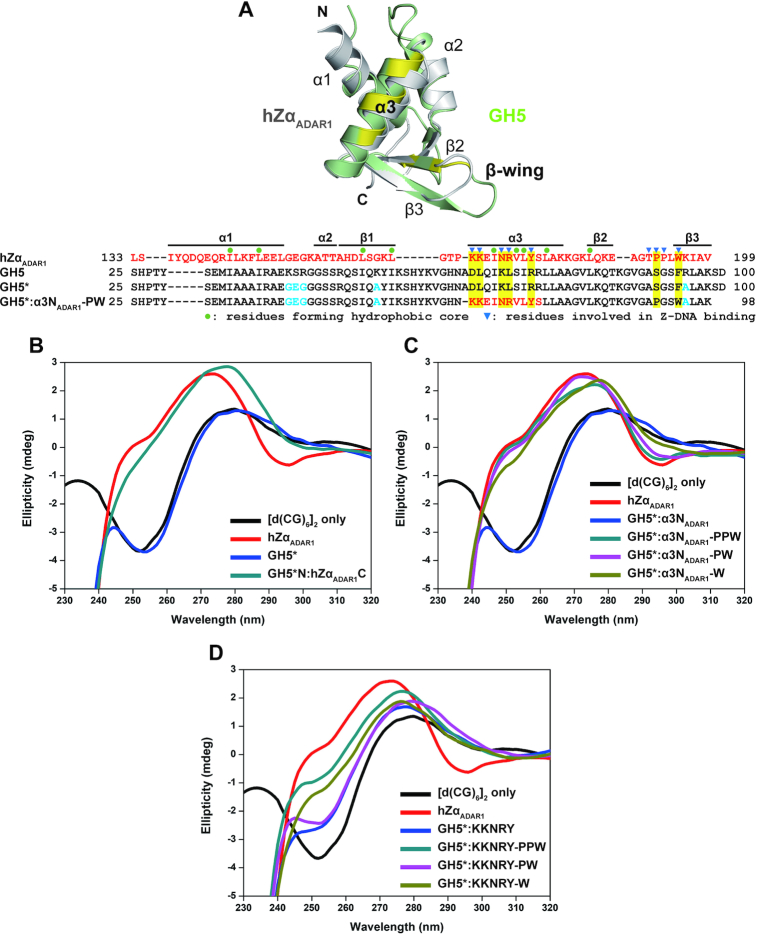
Design of GH5 mutants and their CD spectra. (**A**) Structures of free GH5 (PDB ID 1HST, pale green) and hZα_ADAR1_ (PDB ID 1QBJ, gray) were aligned. Although these two proteins share about 12% sequence identity and have different loop lengths, the overall structures were well aligned, with an RMSD of 1.9 Å. Sequence alignment of hZα_ADAR1_, GH5, GH5*, and GH5*:α3N_ADAR1_-PW is shown below. In the chimeric mutant, residues derived from hZα_ADAR1_ are in red. Mutated residues in GH5* are in cyan. Residues involved in interaction with Z-DNA in hZα_ADAR1_/Z-DNA complex, but not conserved in GH5 and GH5* are highlighted in yellow. CD spectra of the DNA duplex measured in the presence of (**B**) GH5* and GH5*N:hZα_ADAR1_C, (**C**) GH5*:α3N_ADAR1_-series mutants and (**D**) GH5*:KKNRY-series mutants are presented. GH5* has no B–Z transition activity, while its mutants that contain crucial residues for the Z-DNA interaction in the α3 helix and the β-wing show clear B–Z transition activities. The sequences of GH5*N:hZα_ADAR1_C, GH5*:α3N_ADAR1_-series mutants, and GH5*:KKNRY-series mutants are provided in Supplementary Table S1 and Supplementary Figure S10.

We chose GH5, the globular domain of the linker histone H5, as a target B-DNA binder to engineer into a Z-DNA binding protein because a structural analysis of GH5 showed that it has a high structural homology with hZα_ADAR1_ (Figure 4A). However, GH5 formed insoluble aggregates when mixed with DNA, making it impossible to perform an *in vitro* B–Z transition assay. In a previous study of linker histones, mutations of positively charged residues to uncharged ones were shown to overcome this aggregation problem (50). Similarly, we mutated five residues of GH5 (K41G, S42G, R43G, K53A and R95A) to improve the solubility of the GH5/DNA complex (referred to as GH5* hereinafter). As these mutated residues are not located on the Z-DNA binding interface when the GH5 structure was aligned to the Zα domain structure, we reasonably assumed that they would not be involved in Z-DNA binding after engineering. We successfully purified GH5* and performed B–Z transition assays. In the CD spectrum, the DNA duplex containing CG repeats showed a B-conformation of DNA in the presence of GH5*, indicating that GH5* does not have B–Z transition ability (Figure 4B).

To engineer GH5* to be a Z-DNA binder, we first designed a chimeric mutant GH5*N:hZα_ADAR1_C that consists of the N-terminal half (α1–β1–α2) of GH5* and the C-terminal half (α3–β2–β3) of hZα_ADAR1_ (Supplementary Figure S10). This mutant showed a comparable B–Z transition activity to hZα_ADAR1_ (Figure 4B). Thus, we concluded that this chimeric protein is well-folded and presumably has a similar wHTH motif structure to hZα_ADAR1_. Two series of GH5* mutants were constructed to have Z-DNA binding and B-to-Z conversion ability. The first mutant (denoted as GH5*:α3N_ADAR1_) was designed to have the N-terminal part of the α3 helix (K169–S178) of hZα_ADAR1_ substituted for the corresponding region of GH5*. The second mutant was designed to have the key Z-DNA-contacting residues of hZα_ADAR1_ (K169, K170, N173, R174 and Y177) in the α3 helix of GH5* (denoted as GH5*-KKNRY). Subsequently, additional substitutions in the β-wing region were introduced into each mutant. These were denoted as -PPW, -PW and -W, indicating that they contain P192/P193/W195, P192/W195 and W195 of hZα_ADAR1_, respectively (Supplementary Table S1 and Supplementary Figure S10).

B–Z transition activities of these two series of mutants are summarized in Table 3 (Figure 4C and D). The results confirmed the importance of Z-DNA-contacting amino acids located in the β-wing. Among PPW residues in the β-wing, the tryptophan appears to be essential for B–Z transition as expected, since it is the crucial residue for both protein stability and DNA binding by direct interaction with the key tyrosine residue and water-mediated interaction with the phosphate backbone (18,25,31–38). In GH5*, phenylalanine (F94) replaces the tryptophan (W195 in hZα_ADAR1_). Consistently, the W195F mutation of hZα_ADAR1_ was shown to reduce B–Z transition activity significantly (unpublished data), suggesting that phenylalanine cannot fully replace the tryptophan residue in this position.

**Table 3.** Relative B–Z transition activity of each GH5* mutant compared to that of hZα_ADAR1_

Protein	B–Z conversion (%)
hZα_ADAR1_	100±3.8^a^
GH5*	0±0.1
GH5*N:hZα_ADAR1_C	91±3.4
GH5*:α3N_ADAR1_	0±0.0
GH5*:α3N_ADAR1_-W	87±3.3
GH5*:α3N_ADAR1_-PW	94±3.6
GH5*:α3N_ADAR1_-PPW	97±3.7
GH5*-KKNRY	28±1.1 (37±1.4^b^)
GH5*-KKNRY-W	61±2.3
GH5*-KKNRY-PW	31±1.2 (58±2.1^b^)
GH5*-KKNRY-PPW	73±2.8

^a^The relative B–Z transition activity compared to that of hZα_ADAR1_ at a [P]/[N] ratio of 4.

^b^The numbers in parentheses indicate the B–Z transition activity at a [P]/[N] ratio of 30.

In our effort to create a GH5* mutant that enables B–Z transition with minimal substitutions, two mutants are noteworthy. One is GH5*-KKNRY-PPW, which has 8 substituted amino acids (K169, K170, N173, R174, Y177, P192, P193 and W195 of hZα_ADAR1_) that are essential for Z-DNA contacts based on the structure of the Zα/Z-DNA complex (18,25,31–38). This mutant has a good B–Z transition activity (Figure 4D and Table 3). The other mutant, GH5*-KKNRY-W, having K169, K170, N173, R174, Y177 and W195 of hZα_ADAR1_, showed substantial B–Z transition activity despite the absence of two Pro residues (P192 and P193 of hZα_ADAR1_) in the β-wing (Figure 4D and Table 3). Although P192 and P193 (as well as T191) were defined as Z-DNA contacting residues in hZα_ADAR1_, they are deficient in a few Zα family members. Thus, the less conserved Pro residues in the β3 strand do not appear to be essential for the B–Z transition activity. As expected from the importance of highly conserved tryptophan residue, the GH5*-KKNRY lacking W195 in the β3 strand showed much lower B–Z transition activity. Consequently, a Z-DNA binding protein was artificially built with a defined positional display of essential Z-DNA-contacting residues on the backbone of a precisely defined structure that belongs to WHD.

### Z-DNA binding affinity of engineered chimeric GH5

In the previous section, we showed that an α3-swapped vvZα_E3L_ mutant with B–Z transition activity acquired B-DNA binding ability. Similarly, we analyzed Z- and B-DNA binding affinities of GH5*:α3N_ADAR1_-PW, which showed an almost identical B–Z transition CD spectrum to that of hZα_ADAR1_ (Figure 4C). To overcome the aggregation problem during MST, the experiments were carried out in relatively high salt conditions (100 mM NaCl) compared to other experimental conditions for vvZα_E3L_ mutants and hZα_ADAR1_ (20 mM NaCl). As a control, the Z-DNA binding affinity of hZα_ADAR1_ was measured at high salt conditions, and it showed a much higher *K*_D_ value (*K*_D_ of 600 nM) than that measured in the 20 mM NaCl condition (*K*_D_ of 18 nM). The B-DNA binding affinity of hZα_ADAR1_ was reduced 13-fold (*K*_D_ of 35 μM) at high salt conditions, suggesting that the polar interactions of hZα_ADAR1_ are important for Z- and B-DNA binding. MST experiments with GH5*:α3N_ADAR1_-PW showed that this GH5 chimera acquired a Z-DNA binding ability with a *K*_D_ of 1.8 μM, which is just three times lower than that for hZα_ADAR1_ (Table 4 and Supplementary Figure S11). The B-DNA binding affinity of GH5*:α3N_ADAR1_-PW is similar to that of hZα_ADAR1_ with *K*_D_ of 31 μM (Table 4 and Supplementary Figure S11). On the other hand, we also compared the binding affinities of GH5* and GH5*:α3N_ADAR1_-PW with B-DNA using MST to confirm whether or not a change in B-DNA binding ability occurred by engineering of the GH5* chimera from GH5*. An initial trial to measure the B-DNA binding affinity of GH5* under 100 mM NaCl conditions failed due to aggregation during MST. Thus, we performed MST experiments in 150 mM KCl conditions, where the binding affinity between the globular domain of H1 and linear 30 bp DNA was measured previously (51). In this condition, we confirmed that GH5* and GH5*:α3N_ADAR1_-PW have similar binding affinities to B-DNA (Table 5 and Supplementary Figure S12).

**Table 4.** DNA binding affinity of hZα_ADAR1_ and GH5* mutant

Protein	Z-DNA binding affinity (μM)	B-DNA binding affinity (μM)
hZα_ADAR1_	0.60 ± 0.07	35 ± 3
GH5*:α3N_ADAR1_-PW	1.8 ± 0.2	31 ± 2

Experiments were performed at 22°C with a buffer consisting of 20 mM HEPES (pH 8.0), 100 mM NaCl, 0.5 mg/ml BSA, and 0.05% Tween20.

**Table 5.** B-DNA binding affinity of GH5* and GH5* mutant

Protein	B-DNA binding affinity (μM)
GH5*	167 ± 50
GH5*:α3N_ADAR1_-PW	144 ± 16

Experiments were performed at 22°C with a buffer consisting of 20 mM Tris-Cl (pH 7.8), 150 mM KCl, 0.5 mg/ml BSA, and 0.05% Tween20.

Altogether, by mutating the 12 amino acids of GH5*, we successfully engineered GH5*, the B-DNA binder, into a hZα_ADAR1_-like protein with a Z-DNA binding affinity similar to that of hZα_ADAR1_ while maintaining the B-DNA binding ability of GH5*.

### Crystal structure of engineered GH5 in complex with [*d*(TCGCGCG)]_2_

**Figure 5.**
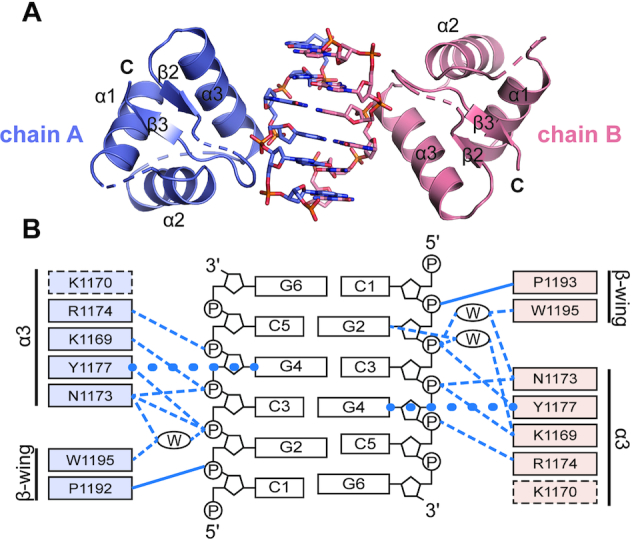
Crystal structure of GH5 mutant in complex with Z-DNA (GH5*:α3N_ADAR1_-PW/Z-DNA). (**A**) The overall structure of GH5*:α3N_ADAR1_-PW complexed with [*d*(TCGCGCG)]_2_ is shown. Chain A and its binding partner DNA are slate colored, chain B and its binding partner DNA are salmon colored. (**B**) Schematic representation of interactions between protein and DNA. Hydrogen bonds are shown as dashed lines, van der Waals contacts are shown as solid lines, and the CH-π interactions are shown as circled lines. Water molecule within the protein–DNA interface is marked with a W inside the oval. Amino acids derived from hZα_ADAR1_ are indicated by adding 1,000 to the amino acid numbers of hZα_ADAR1_ to clarify that those residues are from foreign protein. Residues involved in Z-DNA binding are indicated by colored boxes and the residue, which does not participate in the interaction with Z-DNA is shown by dotted box.

To obtain detailed structural information of the interaction between engineered GH5* chimera and Z-DNA, we determined the crystal structure of one of the B-to-Z converting mutants, GH5*:α3N_ADAR1_-PW, in complex with [*d*(TCGCGCG)]_2_ at a resolution of 2.75 Å (Figure 5A and Supplementary Table S3). Two protein molecules and one duplex DNA molecule, [*d*(TCGCGCG)]_2_, are present in an asymmetric unit of the crystal lattice. In this complex, DNA was shown to form typical Z-conformation with alternating *anti*- and *syn*-conformations of nucleotides and was structurally well aligned to the hZα_ADAR1_-bound Z-DNA (Supplementary Table S4), demonstrating that this GH5* chimera successfully achieved Z-conformation-specific DNA binding in the same manner as hZα_ADAR1_. However, the structural analysis of Z-DNA in complex with GH5* chimera using the web 3DNA 2.0 program (49), showed that the ‘Rise’ value of each step was constant, unlike the zigzag-shaped graph of the ‘Rise’ values found in other Z-DNA structures (Supplementary Figure S13). This difference may be caused by the slight movement of the C-terminal half of α3 helix and β-wing, which affects the conformation of their interacting phosphate backbones (Supplementary Figure S14).

This GH5* chimera has a conserved Z-DNA binding interface using the engineered α3 helix (Figure 5B). The well-conserved interactions are mediated by N1173, R1174, and Y1177 of the α3 helix of this chimera, which are derived from hZα_ADAR1_ (Supplementary Figure S15). Similar to the numbering system used in the vvZα_E3L_ chimera, the residues derived from hZα_ADAR1_ in the GH5* chimera are numbered by adding 1,000 to their original residue numbers. On the other hand, interactions of T191 and P193 on the β-wing of hZα_ADAR1_ are missing in this GH5* chimera structure, while the P1192-mediated β-wing interaction is maintained (Figure 5B).

**Figure 6.**
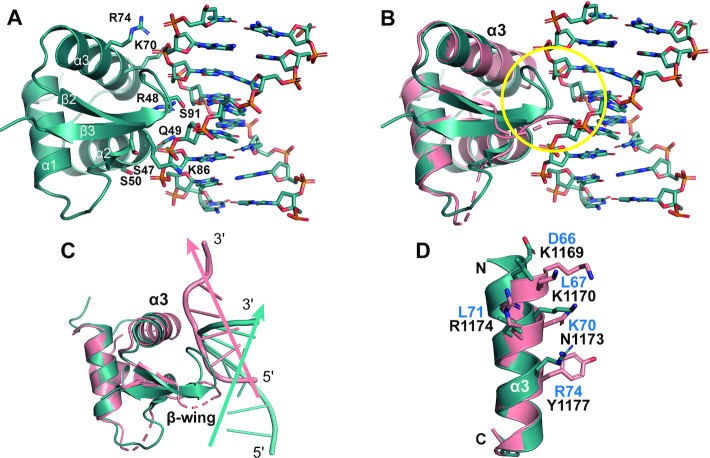
Structural comparison between GH5/B-DNA complex and engineered GH5 mutant/Z-DNA complex. (**A**) The structure of GH5 bound to the phosphate backbone of chromatosome-forming DNA (PDB ID 4QLC (52,53)) is shown. This structure clearly shows the B conformation of DNA. Residues that interact with DNA are represented as sticks (**B**) Structural alignment between GH5*:α3N_ADAR1_-PW (colored in salmon) in complex with Z-DNA and GH5 in complex with B-DNA (colored in teal) reveals steric hindrance between the phosphate backbone of B-DNA and β-wing of GH5*:α3N_ADAR1_-PW, which is marked by a yellow circle. Z-DNA is not shown for clarity. (**C**) Structural alignment between GH5*:α3N_ADAR1_-PW and GH5 indicates different conformations of β-wing and different binding modes for Z-DNA (colored in salmon) and B-DNA (colored in teal). Each arrow represents the central axis of the DNA duplex. The unresolved loop region in the crystal structure is shown as a dashed line. (**D**) Key residues for Z-DNA binding in the α3 helix of GH5*:α3N_ADAR1_-PW (black label) and their corresponding residues of GH5 (blue label) are shown as sticks. It is noteworthy that N1173 and Y1177, which are important for Z-DNA binding in GH5*:α3N_ADAR1_-PW correspond to K70 and R74 of GH5, respectively, which are involved in B-DNA binding.

Whereas the overall structural alignment between GH5*:α3N_ADAR1_-PW and hZα_ADAR1_ gives an RMSD of 2.1 Å, the RMSD value for the alignment of the Z-DNA binding interface including the α3 helix and the β-wing is only 0.80 Å. In contrast, the overall alignment of GH5*:α3N_ADAR1_-PW with free GH5 gives a smaller RMSD value than the local alignment involving only the α3 helix and the β-wing (1.2 Å versus 3.3 Å). This large structural deviation in local alignment is caused by a 19.5° rotation of an α3 helix toward Z-DNA in the GH5*:α3N_ADAR1_–PW/Z–DNA complex structure (Supplementary Figure S16). Introduction of Pro in the S91 position (labeled as P1192 in GH5* chimera) induced the formation of a β-wing structure that facilitates the interaction with P2 of Z-DNA. The charge reversal mutation from D66 to K1169 on an α3 helix may promote the movement of the α3 helix toward the Z-DNA backbone by removing the charge repulsion between Asp and the phosphate backbone and introducing an attractive charge interaction between Lys and P4 (Figure 5B). Mutations from R74 to Y1177 and K70 to N1173 induced specific interactions with the G4 base and P4 of Z-DNA, respectively. Other critical mutations to improve Z-DNA binding include L71 to R1174 and F94 to W1195 mutations, the former interacting with P5, and the latter being important for orienting the Tyr side chain to interact with a G4 base.

Our complex structure of a GH5* chimera demonstrated successful engineering of GH5, a B-DNA binder, into a Z-DNA binder by introducing a few Z-DNA contacting residues on the α3N and in a β-wing, while maintaining the overall structural integrity of the wHTH motif of GH5.

## DISCUSSION

The Zα domain is known to specifically recognize left-handed nucleic acid duplexes including DNA, DNA/RNA hybrid and RNA. Although biochemical and structural studies on Zα domains clearly demonstrate the Z-conformation specific interactions of Zα domains, the molecular mechanism of B–Z transition by the Zα domain is not fully understood. vvZα_E3L,_ the Zα domain of vaccinia viral protein, has an interesting feature in that it binds to Z-DNA using its conserved Z-DNA-interacting residues, but it does not show any B–Z transition activity in physiological conditions even at high excess molar ratios of [P]/[N] (Supplementary Figure S1). In this respect, vvZα_E3L_ is a suitable protein to study B–Z transition activity separately from Z-DNA binding ability. In this study, we successfully transformed vvZα_E3L_ into a functional B-to-Z converter protein by introducing a few point mutations.

This mutational study of vvZα_E3L_ provides two plausible explanations for why vvZα_E3L_ does not have B–Z transition activity. First, vvZα_E3L_ has a relatively loose hydrophobic core, which may cause the key tyrosine residue of the α3 helix to form different rotamers. The vvZα_E3L_-V43I/A46V mutant that could form a more compact hydrophobic core supports this idea because this mutant showed some B–Z transition activity. Apparently, the crystal structure of the vvZα_E3L_ chimeric mutant bound to Z-DNA showed that the key tyrosine residue takes a specific conformation, appropriate for Z-DNA binding, whereas this tyrosine residue showed multiple rotamer conformations in the NMR structure of DNA-free vvZα_E3L_ (Supplementary Figure S17). Second, more importantly, it is conceivable that B-DNA binding may play a significant role in the B–Z transition. We observed that the introduction of positive charges and/or neutralization of a negative charge in the α3C (such as vvZα_E3L_:α3C_ADAR1_ chimera) led to an increased binding affinity for B-DNA. This mutant showed good B–Z transition activity, suggesting that B-DNA binding ability of Z-DNA binding protein is closely correlated with the B–Z transition activity. However, since the vvZα_E3L_:α3C_ADAR1_ mutant also showed enhanced Z-DNA binding affinity, the contribution of Z-DNA stabilization of this mutant to B–Z transition cannot be excluded.

Based on the correlation between B-DNA binding affinity and B–Z transition activity, we postulated that a B-DNA binder could be transformed to be a Zα-like protein if it acquires the Z-DNA binding ability. Using GH5, a B-DNA binder, we created a Zα-like GH5 mutant having both Z-DNA binding and B–Z transition activities by substituting several amino acids located in the α3 helix and β-wing without altering the overall structure of the protein. The engineering of GH5 to a structurally similar but functionally different Zα-like protein was a very intriguing task, especially in terms of changing its ligand conformation specificity. Based on our GH5 engineering results, it appears that structural arrangement of the crucial Z-DNA-contacting residues may be sufficient to have dual-specificity toward the DNA conformation, without the need for modifying the overall structure frame. The crystal structure of the GH5*:α3N_ADAR1_-PW in complex with Z-DNA confirmed that DNA binding proteins with wHTH motifs can be remodeled as a protein with different conformational specificities of DNA while maintaining their overall structures. Consequently, the proteins with a fold wHTH motif such as GH5 could offer a structural basis for creating a novel conformation-specific DNA binding protein. In addition, our result provides an interesting perspective on how nature uses the almost identical tertiary structure to recognize two oppositely-handed conformations of dsDNA. In physiological conditions, Z-DNA formation appears to be dynamic and transient, which would be suitable for a regulatory module to instruct momentary and timely controls over biological processes such as gene expression and genetic control of metabolic networks. By extending our study, it would be interesting to create sequence-specific Z-DNA binders that enrich the usefulness of Z-DNA binders as Z-DNA-based gene expression circuits and control devices for therapeutic and synthetic biological tools.

We showed that the Zα domain with a B–Z transition activity has B-DNA binding ability, but the molecular mechanism of B-DNA binding involved in the B–Z transition process is not clear. Since structural information of the B-DNA-bound Zα domain is not available, we could not directly compare Z-DNA binding interface of Zα domain with its B-DNA binding interface. However, using the crystal structures of chromatosome containing B-DNA-bound GH5 (PDB ID 4QLC and 5WCU) (52,53), it is possible to compare the B-DNA binding mode of GH5 with Z-DNA binding mode of the GH5* chimera. Chromatosome structures showed that GH5 had three binding interfaces, one of which involves α3 and the β-wing, similar to the binding interface between Zα protein and Z-DNA (Figure 6A). When this binding interface was compared to that between the GH5* chimera and Z-DNA, three major differences in the DNA binding mode were observed. First, when GH5*:α3N_ADAR1_-PW is aligned to GH5, the most noticeable difference is the β-wing conformation (Figure 6B). Although GH5*:α3N_ADAR1_-PW has a shorter β-wing, it exhibits steric hindrance to the B-DNA phosphate backbone if it binds to B-DNA in the same mode as observed in the structure of the GH5/B-DNA complex. When GH5 is in complex with B-DNA, the bent β-wing facilitates binding of GH5 to both strands of DNA. In contrast, GH5*:α3N_ADAR1_-PW binds to one strand of Z-DNA duplex. Second, when the GH5/B-DNA complex and GH5*:α3N_ADAR1_–PW/Z-DNA complex are aligned with respect to the protein, the DNA strands bound to the α3 helix are in different directions (Figure 6C). Finally, the GH5 residues corresponding to the major Z-DNA binding residues of GH5*:α3N_ADAR1_-PW are hydrophobic or negatively charged residues except for K70 and R74, which correspond to N1173 and Y1177 of GH5*:α3N_ADAR1_-PW, respectively (Figure 6D). This sequence difference prevents GH5 from interacting with the phosphate backbone of Z-conformation. Multiple B-DNA binding interfaces such as those found in the GH5/B-DNA complexes may promote the large structural transition of the DNA backbones, for example, by bending DNA or recruiting multiple Zα proteins. Indeed, the CD data showed that the B–Z transition increases with increasing [P]/[N] ratios before reaching saturation, suggesting the possibility for multiple Zα proteins to bind to B-DNA.

As the first step in the B–Z transition, the Zα proteins bind to B-DNA through the positive or polar residues, followed by a large structural transition of the DNA phosphate backbones. Although it is highly speculative at present, the initial B-DNA interaction with Zα protein may promote partial melting of DNA duplex through base-pair openings. Base pair rotation then occurs to adopt Z-conformation. Previously, it was proposed that base-pair opening is a crucial step for the B–Z transition process (4) and our previous data also implied that the Zα-induced B–Z transition is dependent on base-pair opening (54). Thus, during the B–Z transition process, many conformational variants of DNA, including a partially unpaired DNA may be produced. In this regard, the CD profiles of DNA obtained using the Zα mutants in this study suggest an interesting aspect of the Zα-induced B–Z transition. The CD spectra of DNA in the presence of weak B–Z converters such as vvZα_E3L_:α3N_ADAR1_ differ noticeably from the canonical CD spectrum of Z-DNA (Supplementary Figure S1). In general, it is assumed that all DNAs would adopt Z-conformation under saturation condition when induced by the strong B–Z converters such as hZα_ADAR1_. On the other hand, the weak B–Z converters, even if excess concentrations are present, do not seem to convert all DNAs to Z-conformation at equilibrium. Thus, the CD spectra of DNA produced by the weak B–Z converters under saturation condition may represent B-DNAs and Z-DNAs as well as partially unpaired DNAs and even single-stranded DNAs stabilized by proteins. At the moment, there is no direct evidence to support our interpretation. Additional studies should be conducted to understand the molecular details of the B–Z transition reaction after B-DNA binding of Zα protein and to interpret this distinguished CD spectral changes.

Overall, our results from the engineering of the two opposite conformation-specific binding proteins, vvZα_E3L_ and GH5, suggest the following conclusion. Paradoxically, Zα-like Z-DNA binding proteins do not rule out B-DNA binding. Rather, engagement with B-DNA may be needed to promote the B–Z transition. How do Zα-like Z-DNA binding proteins then bind to B-DNA? Among diverse DNA binding modes of WHDs, one possibility is that the recognition helix of Zα-like Z-DNA binding proteins could bind at the B-DNA minor groove as observed in the DNA binding domain of human RFX1 transcription factor (55). For now, there are not many clues related to the B-DNA binding mode of Zα-like Z-DNA binding proteins. A study of the complex structure between B-DNA and a Zα-like Z-DNA binding protein may answer this interesting question.

## DATA AVAILABILITY

Atomic coordinates and structure factors for the reported crystal structures have been deposited at the Protein Data Bank under accession numbers 7C0I (vvZα_E3L_:α3_ADAR1_/Z-DNA) and 7C0J (GH5*:α3N_ADAR1_-PW/Z-DNA). Other data used in this work are available from the corresponding author upon reasonable request.

## Supplementary Material

gkaa1115_Supplemental_File
