# Supplementary material for: Dual conformational recognition by Z-DNA binding protein is important for the B–Z transition process
Source: Nucleic Acids Res. 2020 Nov 27;48(22):12957–71. doi: 10.1093/nar/gkaa1115 (PMC7736808; doi:10.1093/nar/gkaa1115)
Supplement: gkaa1115_Supplemental_File [file gkaa1115_supplemental_file.pdf]

Supplementary materials

Dual conformational recognition by Z-DNA binding protein is important for the B-Z transition process

C. Park et al.

Table S1, S2, S3, S4

Figure S1 to S17

Table S1. Construct information.

Construct name	Construct information
hZα$_{ADAR1}$	Human ADAR1 Zα domain 133-199
vvZα$_{E3L}$	vaccinia virus E3L Zα domain 2-78
vvZα$_{E3L}$:α3$_{ADAR1}$	replacement of 40-55 of vvZα$_{E3L}$ with hZα$_{ADAR1}$ 169-184 (α3)
vvZα$_{E3L}$:α3N$_{ADAR1}$	replacement of 40-48 of vvZα$_{E3L}$ with hZα$_{ADAR1}$ 169-177 (α3N)
vvZα$_{E3L}$:α3C$_{ADAR1}$	replacement of 48-55 of vvZα$_{E3L}$ with hZα$_{ADAR1}$ 177-184 (α3C)
vvZα$_{E3L}$-D49S	vvZα$_{E3L}$ with mutation of D49S
vvZα$_{E3L}$-D49R	vvZα$_{E3L}$ with mutation of D49R
vvZα$_{E3L}$-S53K	vvZα$_{E3L}$ with mutation of S53K
vvZα$_{E3L}$-M55K	vvZα$_{E3L}$ with mutation of M55K
vvZα$_{E3L}$-S53K/M55K	vvZα$_{E3L}$ with mutations of S53K and M55K
vvZα$_{E3L}$-D49S/S53K/M55K	vvZα$_{E3L}$ with triple mutations of D49S, S53K, and M55K
vvZα$_{E3L}$-D49R/S53K/M55K	vvZα$_{E3L}$ with triple mutations of D49R, S53K, and M55K
vvZα$_{E3L}$-V43I	vvZα$_{E3L}$ with mutation of V43I
vvZα$_{E3L}$-A46V	vvZα$_{E3L}$ with mutation of A46V
vvZα$_{E3L}$-V43I/A46V	vvZα$_{E3L}$ with mutations of V43I and A46V
vvZα$_{E3L}$-A46V/M53K	vvZα$_{E3L}$ with mutations of A46V and M53K
vvZα$_{E3L}$-A46V/S53K/M55K	vvZα$_{E3L}$ with triple mutations of A46V, S53K, and M55K
GH5	Globular domain of chicken histone H5 25-100
GH5*	GH5 with mutations of K41G, S42G, R43G, K53A, and R95A
GH5*N:hZα$_{ADAR1}$C	Chimera consisting of N-terminal domain of GH5* (25-61) and C-terminal domain of hZα$_{ADAR1}$ (166-199)
GH5*:α3N$_{ADAR1}$	Replacement of 66-75 of GH5* with hZα$_{ADAR1}$ 169-178, and deletions of 89-90 and 99-100
GH5*:α3N$_{ADAR1}$-W	GH5*:α3N$_{ADAR1}$ with mutation of F94W
GH5*:α3N$_{ADAR1}$-PW	GH5*:α3N$_{ADAR1}$ with mutations of S91P and F94W
GH5*:α3N$_{ADAR1}$-PPW	GH5*:α3N$_{ADAR1}$ with mutations of S91P, G92P, and F94W
GH5*-KKNRY	GH5* with mutations of D66K, L67K, K70N, L71R, and R74Y and deletions of 89-90, and 99-100
GH5*-KKNRY-W	GH5*-KKNRY with mutation of F94W
GH5*-KKNRY-PW	GH5*-KKNRY with mutations of S91P and F94W
GH5*-KKNRY-PPW	GH5*-KKNRY with mutations of S91P, G92P, and F94W

Sequences are also shown in Supplementary Figure S2 and S10.

Table S2. Structural statistics of vvZα$_{E3L}$ mutant in complex with Z-DNA.

	vvZα$_{E3L}$:α3$_{ADAR1}$ with [d(TCGCGCG)]$_2$
**Data collection**	
wavelength (Å)	0.9794
Space group	P 3_2 1 2
Unit cell parameters (a, b, c, α, β, γ)	72.759 72.759 123.876 90 90 120
Resolution (Å) (last shell)	31.51 - 2.40 (2.468 - 2.40)
Unique reflections	14876 (1473)
Completeness (%)	99.46 (98.66)
Multipicity	4.5 (4.6)
I/σ(I)	17.94 (2.79)
R_{merge}[a]	0.063 (0.420)
$CC_{1/2}$[b]	0.97 (0.883)
**Refinement**	
No. of reflections working set (test set)	14875 (759)
R_{cryst}/ R_{free}[c]	0.2017 / 0.2274
bond length rmsd from ideal (Å)	0.002
bond angle rmsd from ideal (°)	0.45
Ramachandran analysis[d]	
% favored regions	99.46
% allowed regions	0.54
% outliers	0.00

[a] R_{merge}=ΣhΣl|Iih<Ih>|ΣhΣi(h), where Ii(h) is the ith measurement of reflection h, and <I(h)> is the weighted mean of all measurements of h.

[b] $CC_{1/2}$: Pearson correlation coefficient between random half-datasets (Diederichs & Karplus, 2013).

[c] $R = \Sigma_h|F_{obs}(h)| - |F_{calc}(h)| \,|\, / \Sigma_h|F_{obs}(h)|$. R_{cryst} and R_{free} were calculated using the working and test reflection sets, respectively.

[d]As defined in MolProbity

Table S3. Structural statistics of GH5* mutant in complex with Z-DNA.

	GH5*:α3N$_{ADAR1}$-PW with [d(TCGCGCG)]$_2$
**Data collection**	
wavelength (Å)	0.9794
Space group	P 1 2$_1$ 1
Unit cell parameters (a, b, c, α, β, γ)	31.135 47.305 57.118 90 98.409 90
Resolution (Å) (last shell)	28.25 - 2.75 (2.848 - 2.75)
Unique reflections	4365 (349)
Completeness (%)	96.18 (78.43)
Multipicity	3.7 (3.6)
I/σ(I)	15.03 (3.73)
R_{merge}[a]	0.096 (0.410)
$CC_{1/2}$[b]	0.995 (0.882)
**Refinement**	
No. of reflections working set (test set)	4207 (211)
R_{cryst}/ R_{free}[c]	0.2229 / 0.2482
bond length rmsd from ideal (Å)	0.005
bond angle rmsd from ideal (°)	0.70
Ramachandran analysis[d]	
% favored regions	100.00
% allowed regions	0.00
% outliers	0.00

[a] $R_{merge}=\Sigma h \Sigma I|Iih<Ih>|\Sigma h \Sigma i(h)$, where Ii(h) is the ith measurement of reflection h, and $<I(h)>$ is the weighted mean of all measurements of h.

[b] $CC_{1/2}$: Pearson correlation coefficient between random half-datasets (Diederichs & Karplus, 2013).

[c] $R = \Sigma_h|F_{obs}(h)| - |F_{calc}(h)| \mid / \Sigma_h|F_{obs}(h)|$. R_{cryst} and R_{free} were calculated using the working and test reflection sets, respectively.

[d]As defined in MolProbity

Table S4. Strucutral information of Z-DNAs.

	**vvZα$_{E3L}$:α3$_{ADAR1}$ Bound (7C0I)**	**GH5*:α3N$_{ADAR1}$-PW bound (7C0J)**	**hZα$_{ADAR1}$ bound (1QBJ)**	**Free Z-DNA (4FS6)**	**Ideal B-DNA (Coot)**
Base - sugar orientation					
C1	*anti*	*anti*	*anti*	*anti*	*syn*
G2	*syn*	*syn*	*syn*	*syn*	*syn*
C3	*anti*	*anti*	*anti*	*anti*	*syn*
G4	*syn*	*syn*	*syn*	*syn*	*syn*
C5	*anti*	*anti*	*anti*	*anti*	*syn*
G6	*syn*	*syn*	*syn*	*syn*	*syn*
RMSD (Å)					
hZα$_{ADAR1}$ bound [d(TCGCGCG)]$_2$ (1QBJ)	0.62	0.66		0.66	2.5
Free Z-DNA [d(TCGCGCG)]$_2$ (500 mM $CaCl_2$, 4FS6)	0.80	0.61			2.7
Ideal B-DNA [d(TCGCGCG)]$_2$ (generated by coot)	2.1	2.3			

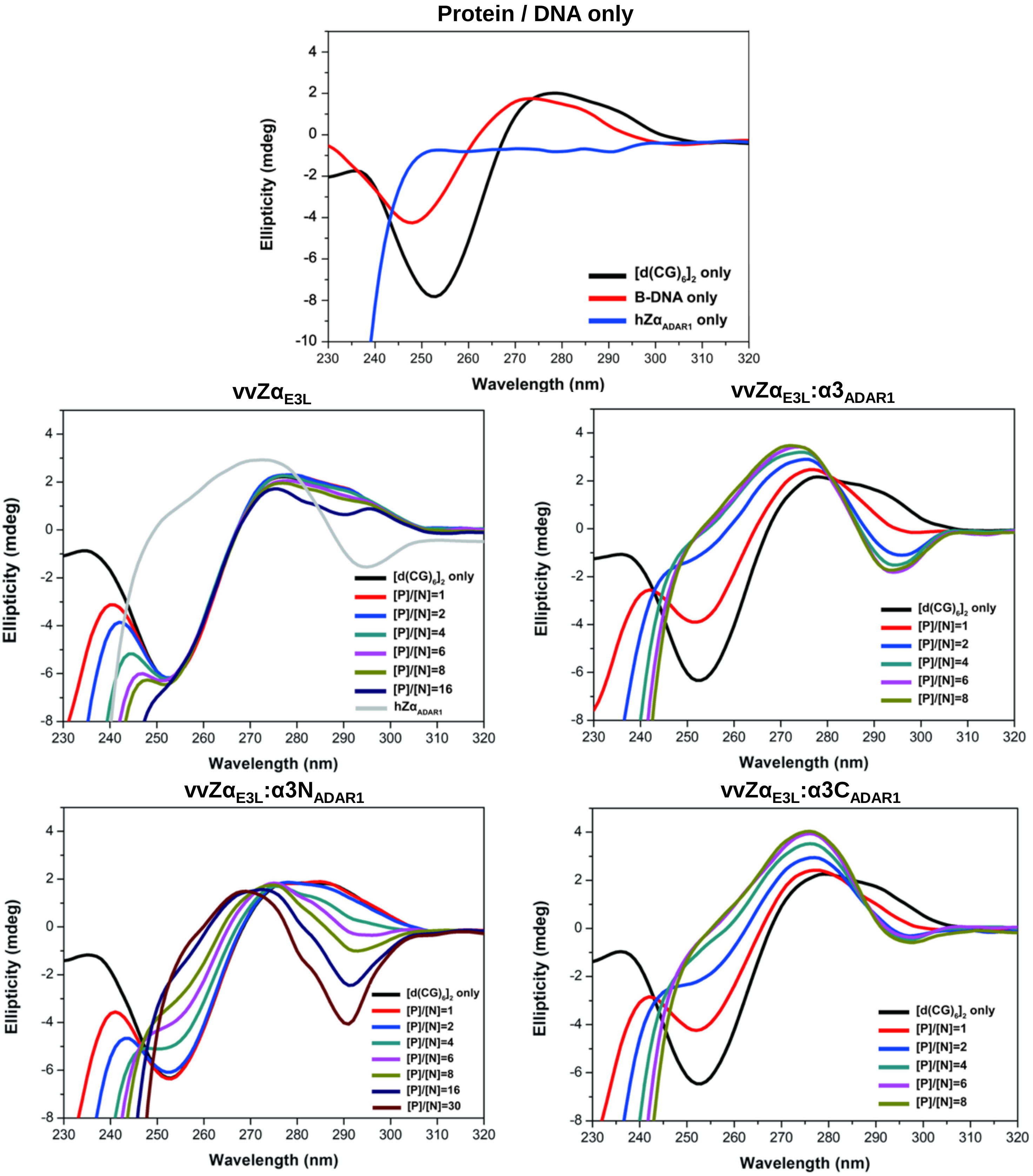

Figure S1. Titration CD spectra of $vvZ\alpha_{E3L}$ and its mutants. As a control, the CD spectra of $hZ\alpha_{ADAR1}$ alone, $[d(CG)_6]_2$ alone, and the B-DNA used for the MST experiment were measured. Control CD experiments clearly show that protein alone does not produce a signal at 255 nm, and $[d(CG)_6]_2$ represents the B-DNA characteristic CD spectrum. $[d(CG)_6]_2$ was titrated with increasing amounts of proteins to obtain a [P]/[N] ratio that reaches saturation for B-Z transition. Clearly, $vvZ\alpha_{E3L}$ does not show B-Z transition even in the presence of excess protein. In contrast, $vvZ\alpha_{E3L}$ mutants induce changes in the CD signals of $[d(CG)_6]_2$. $vvZ\alpha_{E3L}$:$\alpha3_{ADAR1}$ and $vvZ\alpha_{E3L}$:$\alpha3C_{ADAR1}$ reach saturation at 4 [P]/[N], whereas $vvZ\alpha_{E3L}$:$\alpha3N_{ADAR1}$ needs a 16 or higher [P]/[N] ratio to reach saturation. Interestingly, although $vvZ\alpha_{E3L}$:$\alpha3N_{ADAR1}$ altered the CD spectrum of B-DNA, it produced distinct CD spectrum at the saturation condition, characteristically having a small positive peak at 270 nm region and a large spectral reduction at 292 nm region.

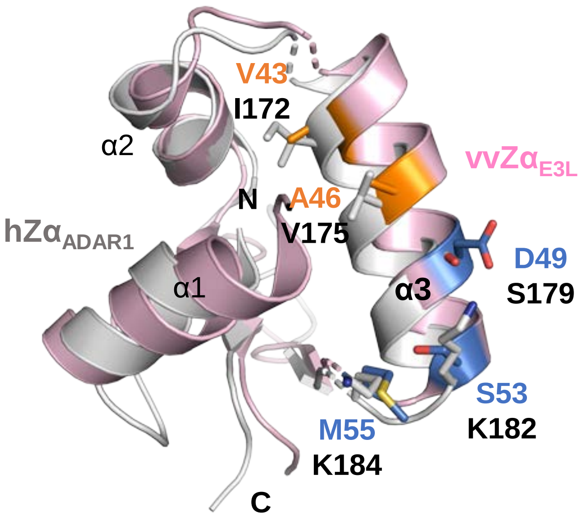

α1 β1 α2 α3 β2 β3

hZα$_{ADAR1}$	133	LS--------IYQDQEQRILKFLEELGEGKATTAHDLSGKLGTPKKEINRVLYSLAKKGKLQKE-AGTPPLWKIAV	199
vvZα$_{E3L}$	2	SKIYIDERSN-----AEIVCEAIKTIGIEG-ATAAQLTRQLNMEKREVNKALYDLQRSAMVYS-SDDIPPRWFMTTEADEADAD	78
vvZα$_{E3L}$:α3$_{ADAR1}$	2	SKIYIDERSN-----AEIVCEAIKTIGIEG-ATAAQLTRQLNMEKKEINRVLYSLAKKGKVYS-SDDIPPRWFMTTEADEADAD	78
vvZα$_{E3L}$:α3N$_{ADAR1}$	2	SKIYIDERSN-----AEIVCEAIKTIGIEG-ATAAQLTRQLNMEKKEINRVLYDLQRSAMVYS-SDDIPPRWFMTTEADEADAD	78
vvZα$_{E3L}$:α3C$_{ADAR1}$	2	SKIYIDERSN-----AEIVCEAIKTIGIEG-ATAAQLTRQLNMEKREVNKALYSLAKKGKVYS-SDDIPPRWFMTTEADEADAD	78
vvZα$_{E3L}$-D49S	2	SKIYIDERSN-----AEIVCEAIKTIGIEG-ATAAQLTRQLNMEKREVNKALYSLQRSAMVYS-SDDIPPRWFMTTEADEADAD	78
vvZα$_{E3L}$-D49R	2	SKIYIDERSN-----AEIVCEAIKTIGIEG-ATAAQLTRQLNMEKREVNKALYRLQRSAMVYS-SDDIPPRWFMTTEADEADAD	78
vvZα$_{E3L}$-S53K	2	SKIYIDERSN-----AEIVCEAIKTIGIEG-ATAAQLTRQLNMEKREVNKALYDLQRKAMVYS-SDDIPPRWFMTTEADEADAD	78
vvZα$_{E3L}$-M55K	2	SKIYIDERSN-----AEIVCEAIKTIGIEG-ATAAQLTRQLNMEKREVNKALYDLQRSAKVYS-SDDIPPRWFMTTEADEADAD	78
vvZα$_{E3L}$-S53K/M55K	2	SKIYIDERSN-----AEIVCEAIKTIGIEG-ATAAQLTRQLNMEKREVNKALYDLQRKAKVYS-SDDIPPRWFMTTEADEADAD	78
vvZα$_{E3L}$ -D49S/S53K/M55K	2	SKIYIDERSN-----AEIVCEAIKTIGIEG-ATAAQLTRQLNMEKREVNKALYSLQRKAKVYS-SDDIPPRWFMTTEADEADAD	78
vvZα$_{E3L}$ -D49R/S53K/M55K	2	SKIYIDERSN-----AEIVCEAIKTIGIEG-ATAAQLTRQLNMEKREVNKALYRLQRKAKVYS-SDDIPPRWFMTTEADEADAD	78
vvZα$_{E3L}$-V43I	2	SKIYIDERSN-----AEIVCEAIKTIGIEG-ATAAQLTRQLNMEKREINKALYDLQRSAMVYS-SDDIPPRWFMTTEADEADAD	78
vvZα$_{E3L}$-A46V	2	SKIYIDERSN-----AEIVCEAIKTIGIEG-ATAAQLTRQLNMEKREVNKVLYDLQRSAMVYS-SDDIPPRWFMTTEADEADAD	78
vvZα$_{E3L}$-V43I/A46V	2	SKIYIDERSN-----AEIVCEAIKTIGIEG-ATAAQLTRQLNMEKREINKVLYDLQRSAMVYS-SDDIPPRWFMTTEADEADAD	78
vvZα$_{E3L}$-A46V/M53K	2	SKIYIDERSN-----AEIVCEAIKTIGIEG-ATAAQLTRQLNMEKREVNKVLYDLQRSAKVYS-SDDIPPRWFMTTEADEADAD	78
vvZα$_{E3L}$ -A46V/S53K/M55K	2	SKIYIDERSN-----AEIVCEAIKTIGIEG-ATAAQLTRQLNMEKREVNKVLYDLQRKAKVYS-SDDIPPRWFMTTEADEADAD	78

●: residues forming hydrophobic core ▼: residues involved in Z-DNA binding

Figure S2. Sequence comparison of hZα$_{ADAR1}$ and vvZα$_{E3L}$ mutants. (Upper) The residues subjected to point mutations are shown as sticks in ribbon diagram. The color of each chain follows the same color code as in **Figure 1A**. N-terminus of the hZα$_{ADAR1}$ α1 helix is omitted for clarity. (Bottom) Sequence alignment of hZα$_{ADAR1}$, vvZα$_{E3L}$, and chimeric and point mutants of vvZα$_{E3L}$ are shown. In vvZα$_{E3L}$ mutants, residues originated from hZα$_{ADAR1}$ are colored red, and green letters indicate charge reversal mutations, which are not from hZα$_{ADAR1}$. Secondary structural elements of hZα$_{ADAR1}$ are shown on top of the sequences.

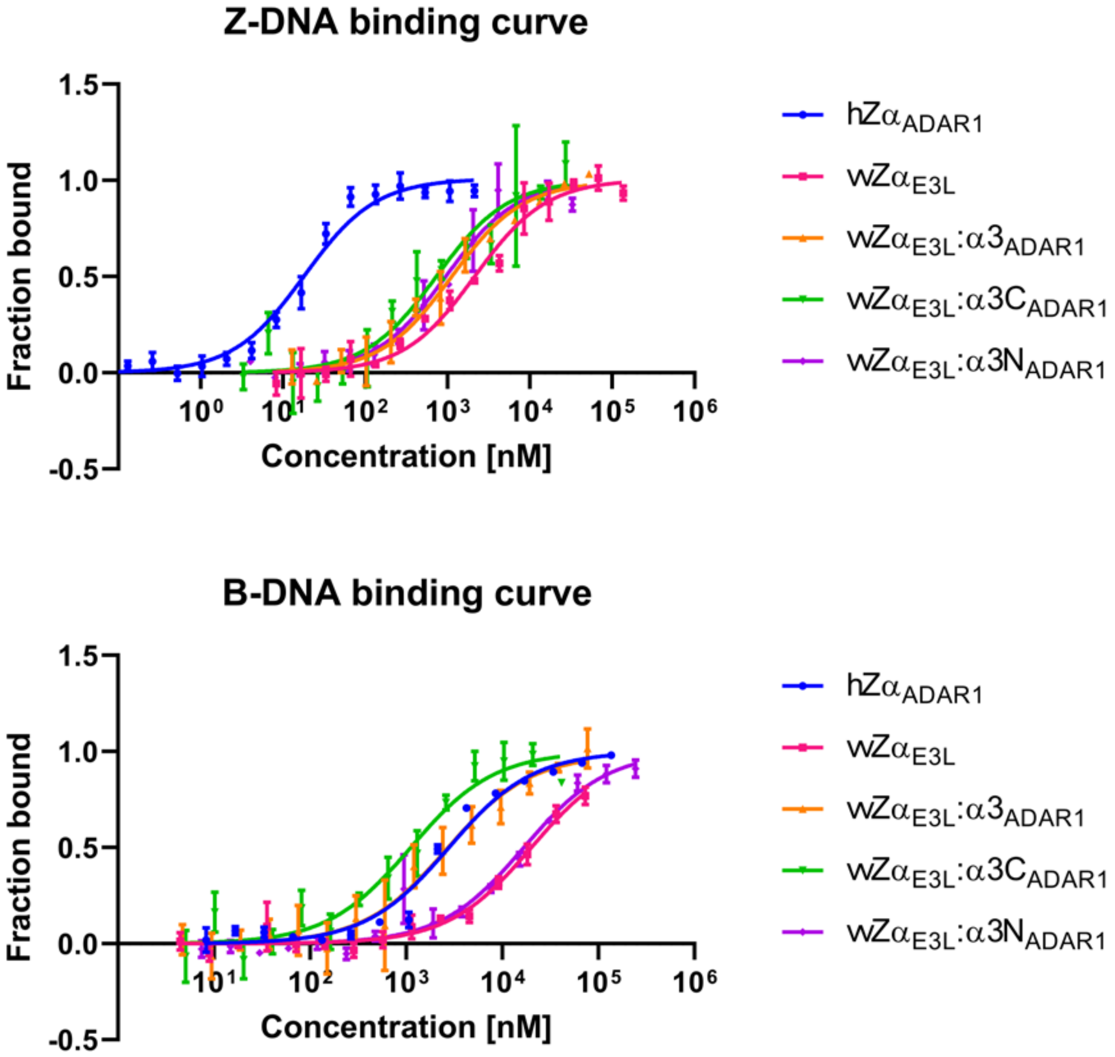

Figure S3. MST data of hZα$_{ADAR1}$ and wild-type and chimeric mutants of vvZα$_{E3L}$. Binding affinity to **c**onformation-specific DNA was measured by microscale thermophoresis (MST), and the representative curves of each independent experiment are shown. (Upper) Z-DNA binding curves of 5 proteins are overlaid. hZα$_{ADAR1}$ has much higher affinity to Z-DNA than any vvZα$_{E3L}$ proteins. Wild-type and α3-swapped mutants of vvZα$_{E3L}$ have similar Z-DNA binding affinity. (Lower) B-DNA binding curves are overlaid. vvZα$_{E3L}$:α3$_{ADAR1}$ and vvZα$_{E3L}$:α3C$_{ADAR1}$ show enhanced B-DNA binding affinity compared to wild-type vvZα$_{E3L}$. Measured K_D values are shown in **Table 2**. Experimental conditions are described in **Material and Methods**. Error bars represent SD of three independent experiments.

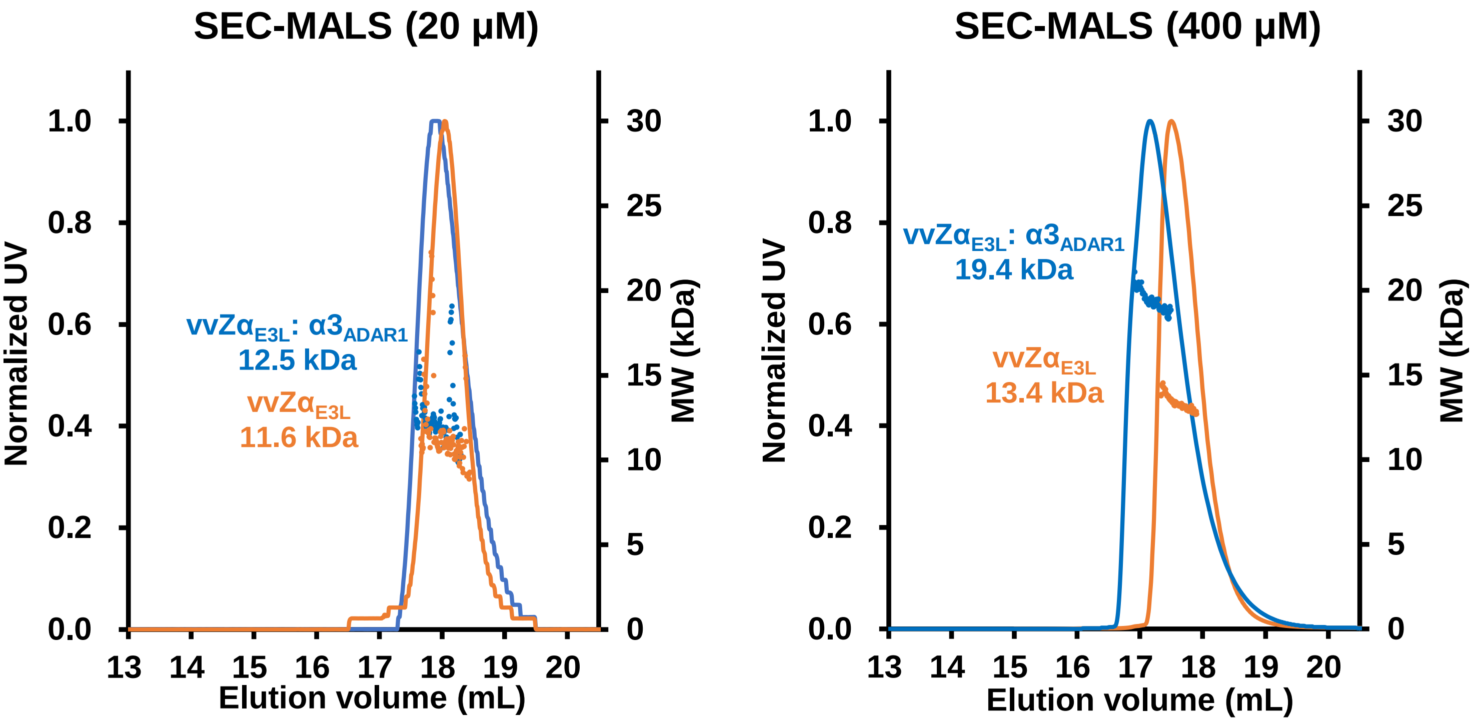

Figure S4. SEC-MALS profiles of vvZα$_{E3L}$ and vvZα$_{E3L}$:α3$_{ADAR1}$ at different injection concentrations. (Left) An overlay of SEC-MALS profiles of vvZα$_{E3L}$ (orange) and vvZα$_{E3L}$:α3$_{ADAR1}$ (blue) at 20 μM concentration is shown. Measured absolute molecular masses of vvZα$_{E3L}$ (orange) and vvZα$_{E3L}$:α3$_{ADAR1}$ (blue) at 20 μM concentration were similar, 11.6 kDa and 12.5 kDa, respectively. Both are bigger than their theoretical values (9.1 kDa for vvZα$_{E3L}$ and 9.0 kDa for vvZα$_{E3L}$:α3$_{ADAR1}$). (Right) An overlay of SEC-MALS profiles of vvZα$_{E3L}$ (orange) and vvZα$_{E3L}$:α3$_{ADAR1}$ (blue) at 400 μM concentration is shown. The absolute molecular mass of vvZα$_{E3L}$:α3$_{ADAR1}$ was measured as 19.4 kDa, which is close to its theoretical value of dimer (18.0 kDa). In contrast, the absolute molecular mass of wild-type vvZα$_{E3L}$ was measured as 13.4 kDa, similar to that measured at 20 μM concentration.

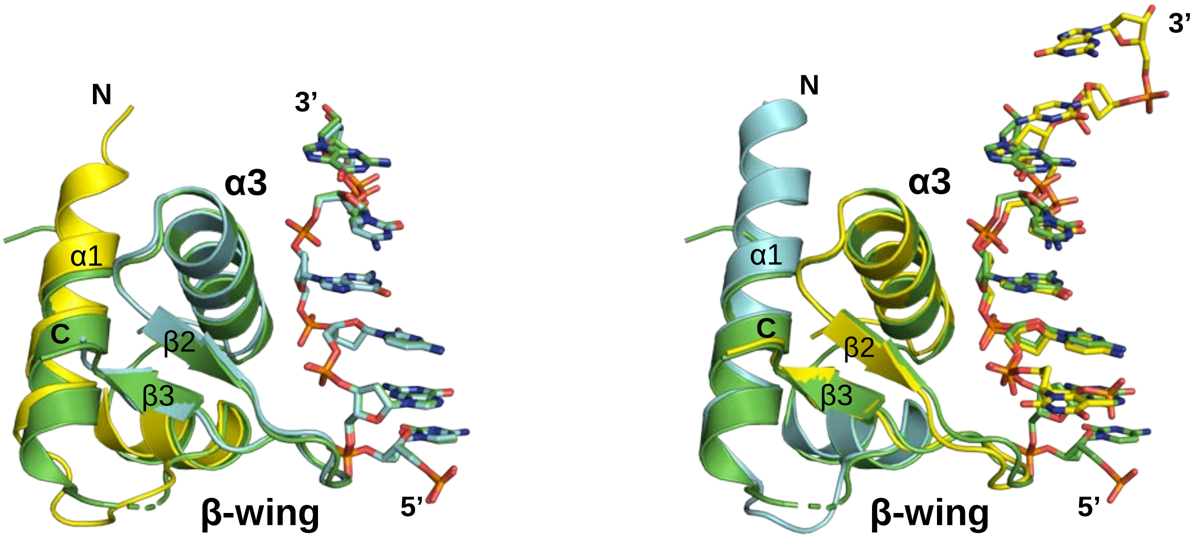

Figure S5. Structural comparison between monomeric and dimeric forms of vvZα$_{\mathbf{E3L}}$:α3$_{\mathbf{ADAR1}}$. (Left) Chain B* and chain E were aligned to chains A and D. (Right) Chain C* and chain F were aligned to chains A and D. Chain B* is a composite model consisting of the C-terminal half of chain B (aa 37-69) and the N-terminal half of chain C (aa 6-36). Chain C* is a composite model consisting of the N-terminal half of chain B (aa 3-36) and the C-terminal half of chain C (aa 37-70). The color of each chain follows the same color code as in **Figure 3**.

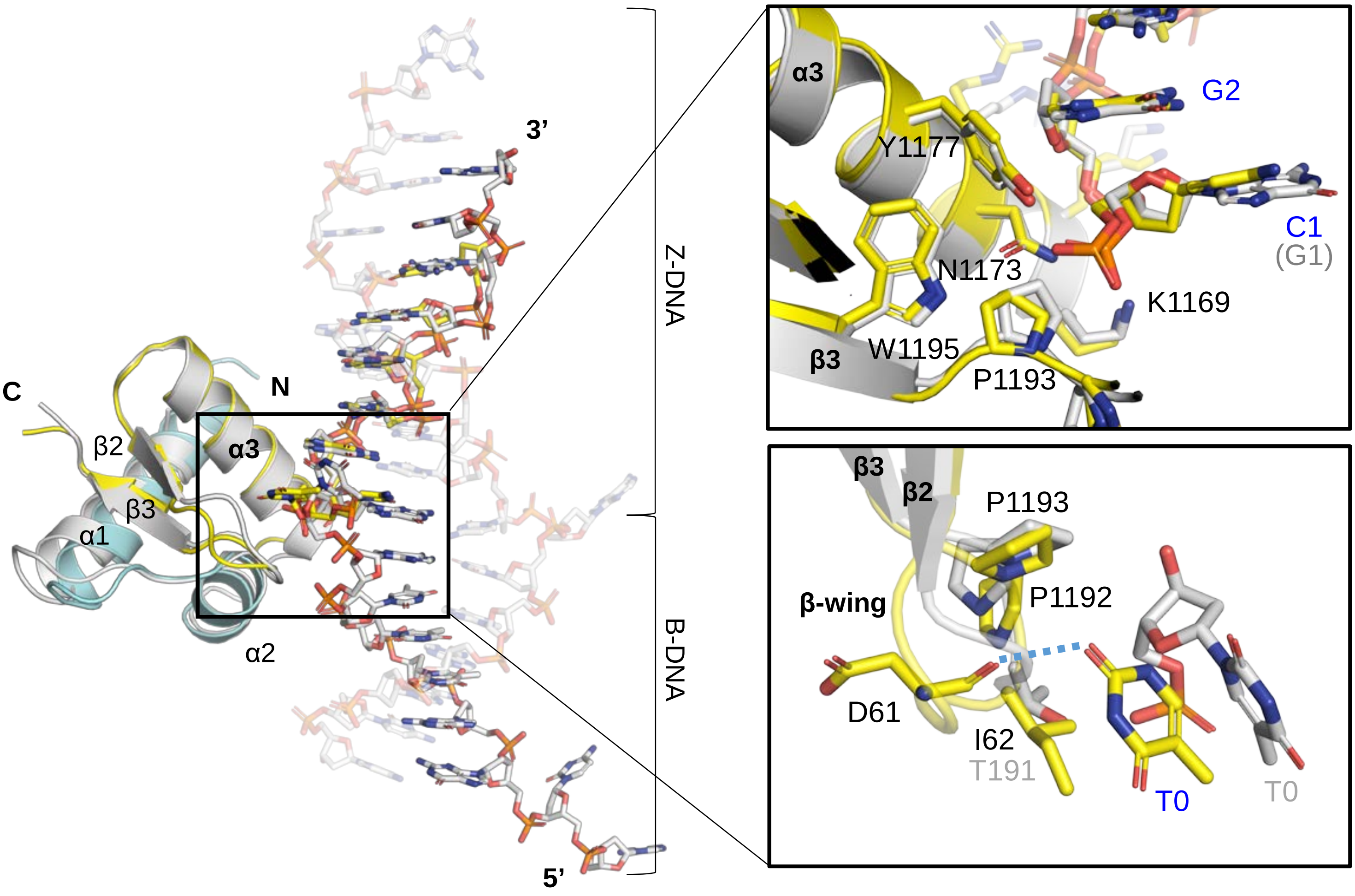

Figure S6. Structural comparison of B-Z junction and vvZα$_{E3L}$ mutant. Chains C* and F of the vvZα$_{E3L}$:α3$_{ADAR1}$/Z-DNA complex are aligned to the corresponding regions of the B-Z junction structure (PDB ID 2ACJ, gray). Chain C* has an unusual Z-DNA binding interface similar to that found in the B-Z junction (magnified view, upper box). A different T0 orientation is shown in the magnified view at the bottom. T0 base is in close contacts with the carbonyl group of D61 and side chains of I62 and P1193 in the vvZα$_{E3L}$:α3$_{ADAR1}$/Z-DNA structure. Polar contact between D61 and T0 is represented as a blue dotted line.

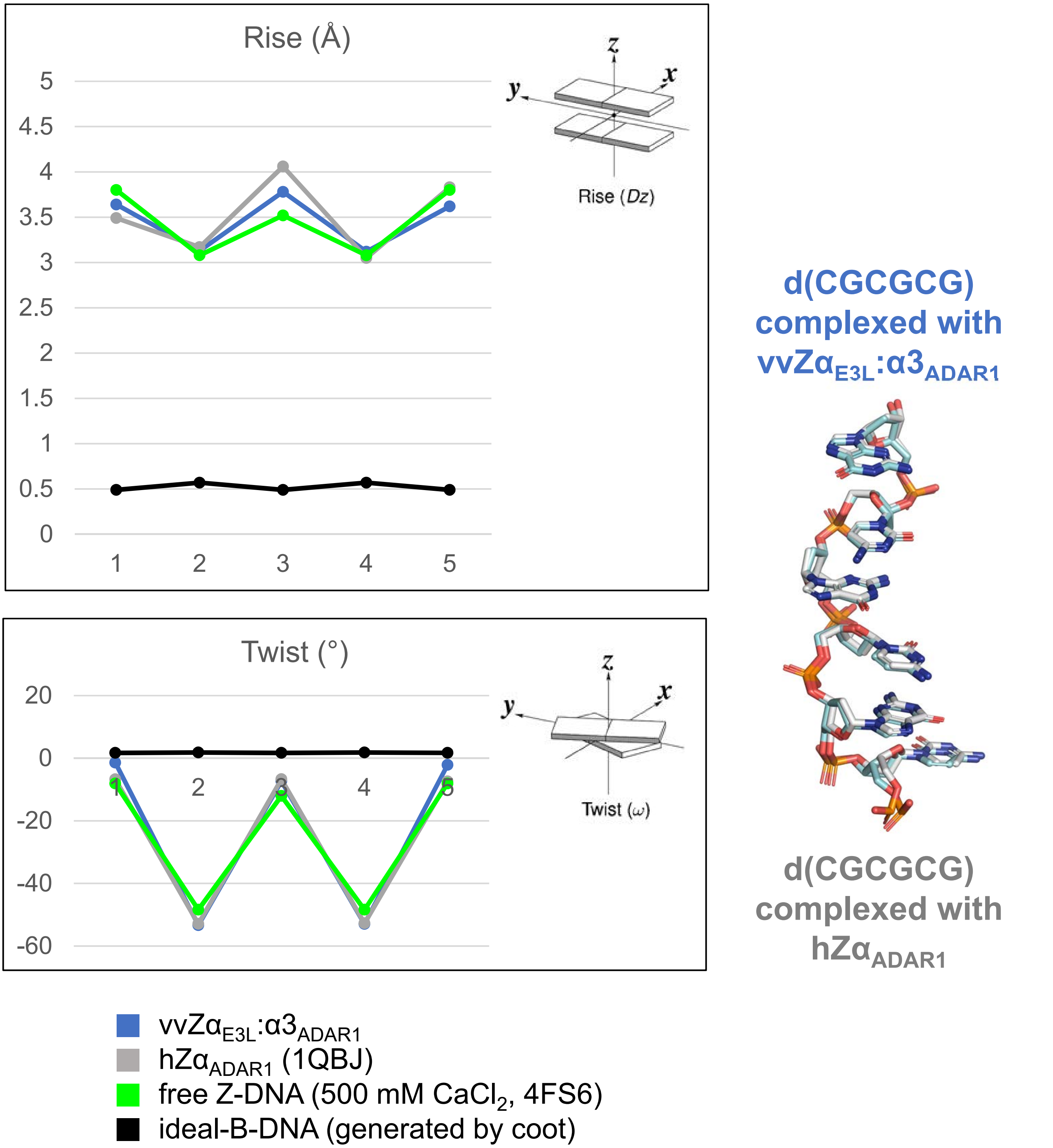

Figure S7. Structural analysis of the Z-DNA duplex from the vvZα$_{E3L}$:α3$_{ADAR1}$/Z-DNA complex. Crystal structure of the Z-DNA duplex from the vvZα$_{E3L}$:α3$_{ADAR1}$/Z-DNA complex (blue), hZα$_{ADAR1}$/Z-DNA complex (PDB ID 1QBJ, gray), free Z-DNA (PDB ID 4FS6, green), and ideal B-DNA generated by Coot (black) are analyzed by web 3DNA 2.0 (http://web.x3dna.org/analyze/). All four structures have the same sequence, CGCGCG. 'Rise' and 'Twist' are the local base-pair step parameters; the former is the distance between each step; the latter indicates the extend of rotation of each step. Both parameters are based on ideal B-DNA, and the two graphs clearly show that d(CGCGCG)$_2$ is different from ideal B-DNA, extended along the z-axis, and negatively twisted (left-handedness). Especially, the 'Rise' at step 3 seems to reflect the conserved interaction between the third and fourth phosphates of Z-DNA and the α3 helix of Zα protein.

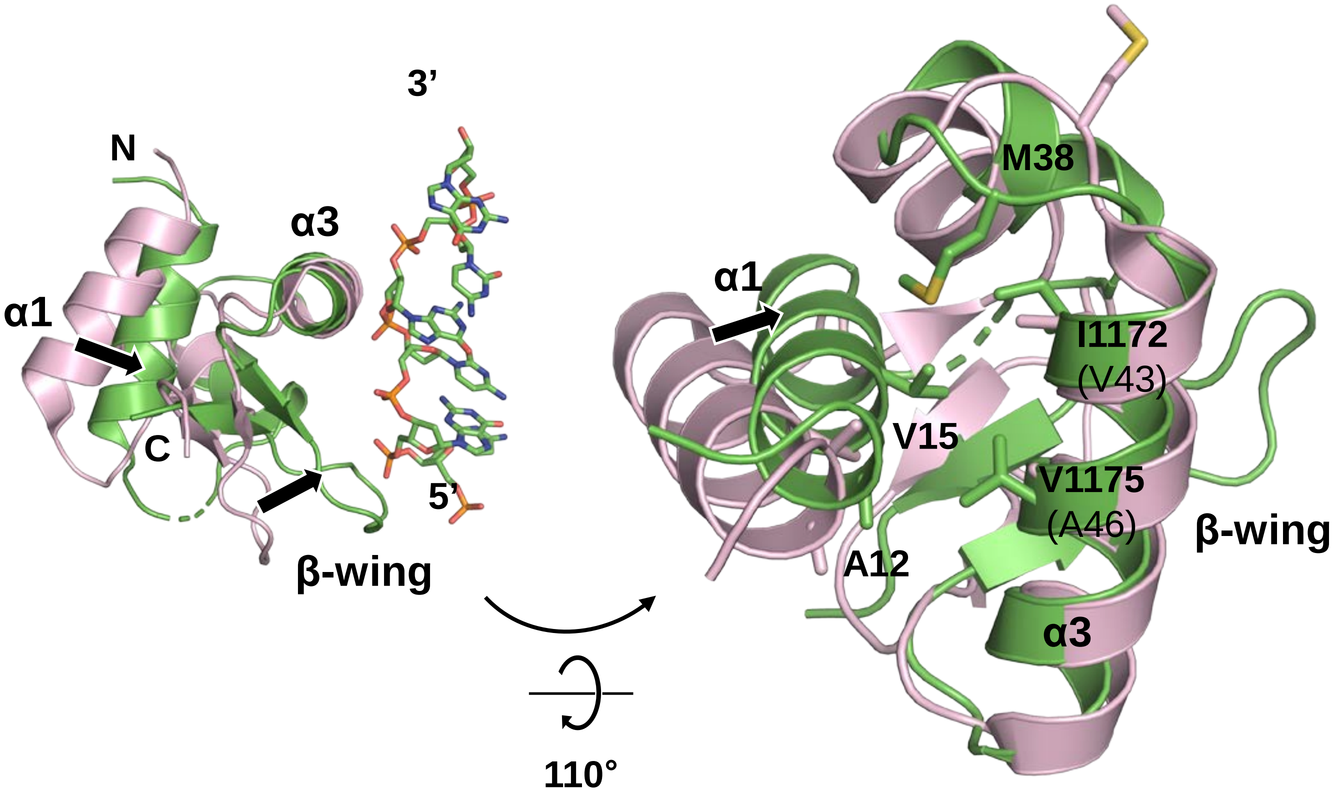

Figure S8. Comparison of the hydrophobic core between free vvZα$_{E3L}$ and vvZα$_{E3L}$ chimeric mutant in complex with Z-DNA. Crystal structure of monomeric vvZα$_{E3L}$:α3$_{ADAR1}$/Z-DNA complex (chains A and D, green) and NMR structure of free vvZα$_{E3L}$ (PDB ID 1OYI, light pink) are aligned against their α3 helices. (Left) Movement of the α1 helix and β-wing is represented with black arrows. (Right) Residues forming hydrophobic core are shown as sticks. In vvZα$_{E3L}$:α3$_{ADAR1}$, amino acids derived from hZα$_{ADAR1}$ are indicated by adding 1,000 to residue numbers of hZα$_{ADAR1}$ and original residues of vvZα$_{E3L}$ in the α3 helix are shown with parenthesis. Z-DNA is omitted for simplicity.

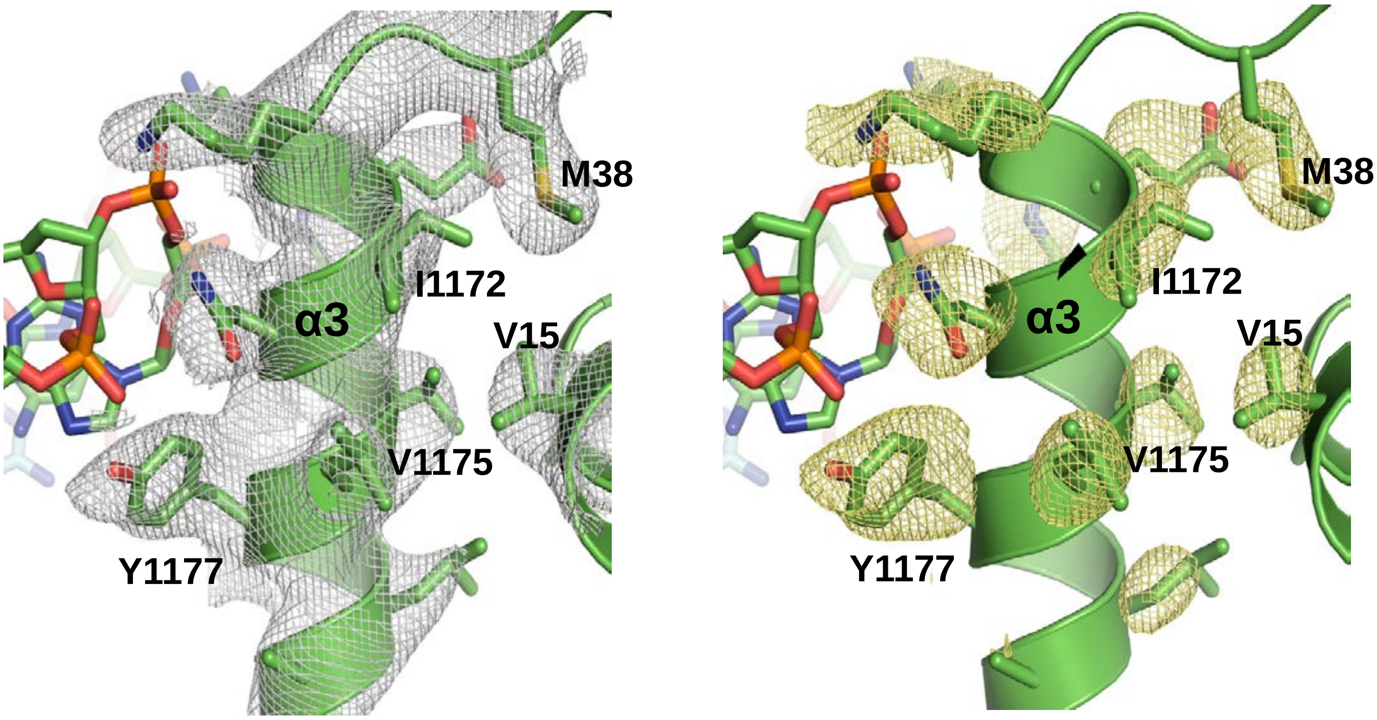

Figure S9. Composite omit map of the vvZα$_{E3L}$:α3$_{ADAR1}$/Z-DNA complex structure. Composite omit maps of 2mFo−DFc (left) and mFo-DFc (right) contoured at 1.0 σ and 3.0 σ levels, respectively, are shown for the α3 helix region of the vvZα$_{E3L}$:α3$_{ADAR1}$/Z-DNA complex. The electron density maps are shown as mesh (gray and yellow). Residues forming a tight hydrophobic core (A12, V15, M38, I1172, V1175) are well defined. The electron density maps confirm the specific rotamer conformation of Y1177.

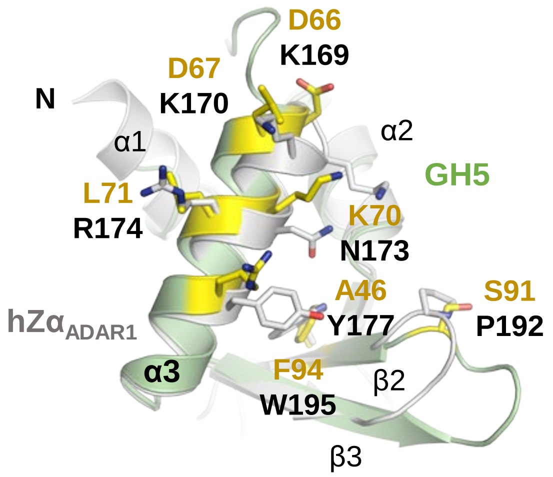


```
                                  α1            β1   α2                 α3            β2          β3
hZαADAR1                   133 LS---IYQDQEQRILKFLEELGEGKATTAHDLSGKL------GTP-KKEINRVLYSLAKKGKLQKE--AGTPPLWKIAV    199
GH5                         25 SHPTY-----SEMIAAAIRAEKSRGGSSRQSIQKYIKSHYKVGHNADLQIKLSIRRLLAAGVLKQTKGVGASGSFRLAKSD 100
GH5*                        25 SHPTY-----SEMIAAAIRAEGEGGGSSRQSIQAYIKSHYKVGHNADLQIKLSIRRLLAAGVLKQTKGVGASGSFALAKSD 100
GH5*N:hZαADAR1C             25 SHPTY-----SEMIAAAIRAEGEGGGSSRQSIQAYIKSHYKVGTP-KKEINRVLYSLAKKGKLQKE--AGTPPLWKIAV
GH5*N:hZαADAR1C:α3NGH5      25 SHPTY-----SEMIAAAIRAEGEGGGSSRQSIQAYIKSHYKVGTP-DLQIKLSLYSLAKKGKLQKE--AGTPPLWKIAV
GH5*N:hZαADAR1C:α3CGH5      25 SHPTY-----SEMIAAAIRAEGEGGGSSRQSIQAYIKSHYKVGTP-KKEINRVLYSLLAAGVLQKE--AGTPPLWKIAV
GH5*:α3NADAR1               25 SHPTY-----SEMIAAAIRAEGEGGGSSRQSIQAYIKSHYKVGHN-KKEINRVLYSLLAAGVLKQTKGVGASGSFALAK    98
GH5*:α3NADAR1-W             25 SHPTY-----SEMIAAAIRAEGEGGGSSRQSIQAYIKSHYKVGHN-KKEINRVLYSLLAAGVLKQTKGVGASGSWALAK    98
GH5*:α3NADAR1-PW            25 SHPTY-----SEMIAAAIRAEGEGGGSSRQSIQAYIKSHYKVGHN-KKEINRVLYSLLAAGVLKQTKGVGAPGSWALAK    98
GH5*:α3NADAR1-PPW           25 SHPTY-----SEMIAAAIRAEGEGGGSSRQSIQAYIKSHYKVGHN-KKEINRVLYSLLAAGVLKQTKGVGAPPSWALAK    98
GH5*:KKNRY                  25 SHPTY-----SEMIAAAIRAEGEGGGSSRQSIQAYIKSHYKVGHN-KKQINRSIYRLLAAGVLKQTKGVGASGSFALAK    98
GH5*:KKNRY-W                25 SHPTY-----SEMIAAAIRAEGEGGGSSRQSIQAYIKSHYKVGHN-KKQINRSIYRLLAAGVLKQTKGVGASGSWALAK    98
GH5*:KKNRY-PW               25 SHPTY-----SEMIAAAIRAEGEGGGSSRQSIQAYIKSHYKVGHN-KKQINRSIYRLLAAGVLKQTKGVGAPGSWALAK    98
GH5*:KKNRY-PPW              25 SHPTY-----SEMIAAAIRAEGEGGGSSRQSIQAYIKSHYKVGHN-KKQINRSIYRLLAAGVLKQTKGVGAPPSWALAK    98
```

●: residues forming hydrophobic core ▼: residues involved in Z-DNA binding

Figure S10. Sequence comparison of hZα$_{ADAR1}$ and GH5 mutants. (Upper) Residues subjected to mutation are shown as sticks in ribbon diagramusing the same color code as in Figure 4A. (Bottom) Sequence alignment of hZα$_{ADAR1}$, GH5, GH5*, and GH5* chimeric mutants are shown. Residues from hZα$_{ADAR1}$ are shown with red letters, and mutated residues in GH5* are highlighted in gray boxes. Secondary structural elements of hZα$_{ADAR1}$ are shown on top of the sequences.

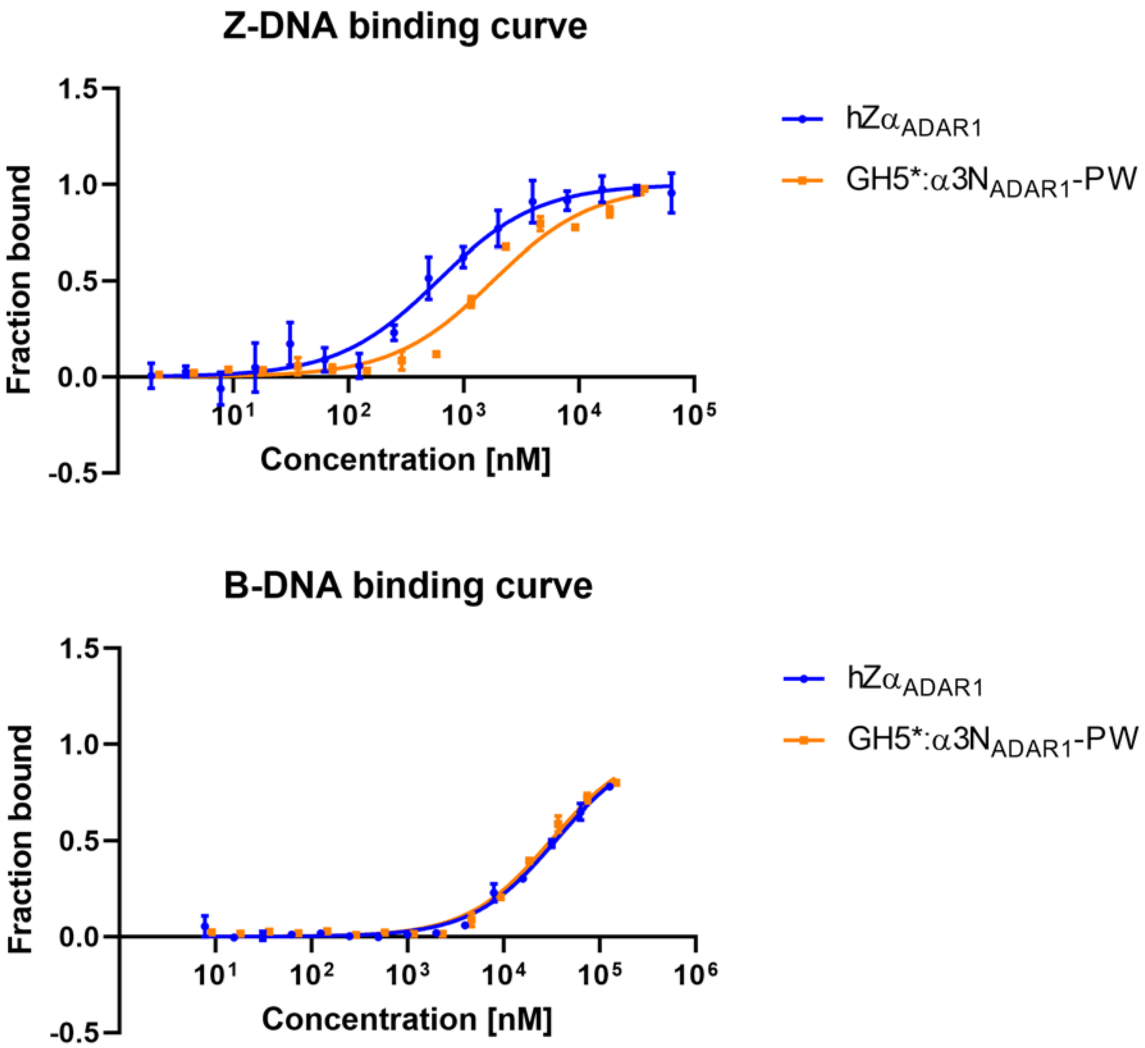

Figure S11. MST data for hZα$_{ADAR1}$ and GH5*:α3N$_{ADAR1}$-PW. Binding affinity to conformation-specific DNA was measured by microscale thermophoresis (MST). (Upper) Z-DNA binding curves of hZα$_{ADAR1}$ and GH5*:α3N$_{ADAR1}$-PW are overlaid. hZα$_{ADAR1}$ has higher affinity to Z-DNA than does the GH5* mutant. (Lower) B-DNA binding curves are overlaid. hZα$_{ADAR1}$ and GH5*:α3N$_{ADAR1}$-PW have similar B-DNA binding affinity. Measured K_D values are shown in Table 4. Experimental conditions are described in Material and Methods. Error bars represent the SD of three independent experiments.

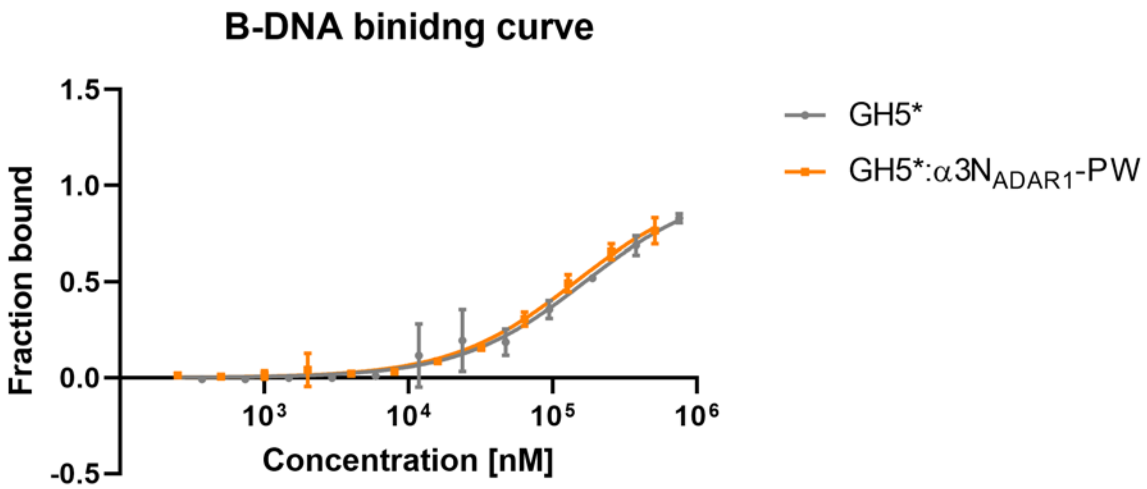

Figure S12. MST data for GH5* and GH5*:α3N$_{ADAR1}$-PW. B-DNA binding curves of GH5* and GH5*:α3N$_{ADAR1}$-PW measured by microscale thermophoresis (MST) are overlaid. GH5*:α3N$_{ADAR1}$-PW and GH5* showed similar B-DNA binding affinity. Measured K_D values are shown in **Table 5**. Experimental condition is described in **Material and Methods**. Error bars represent the SD of three independent experiments.

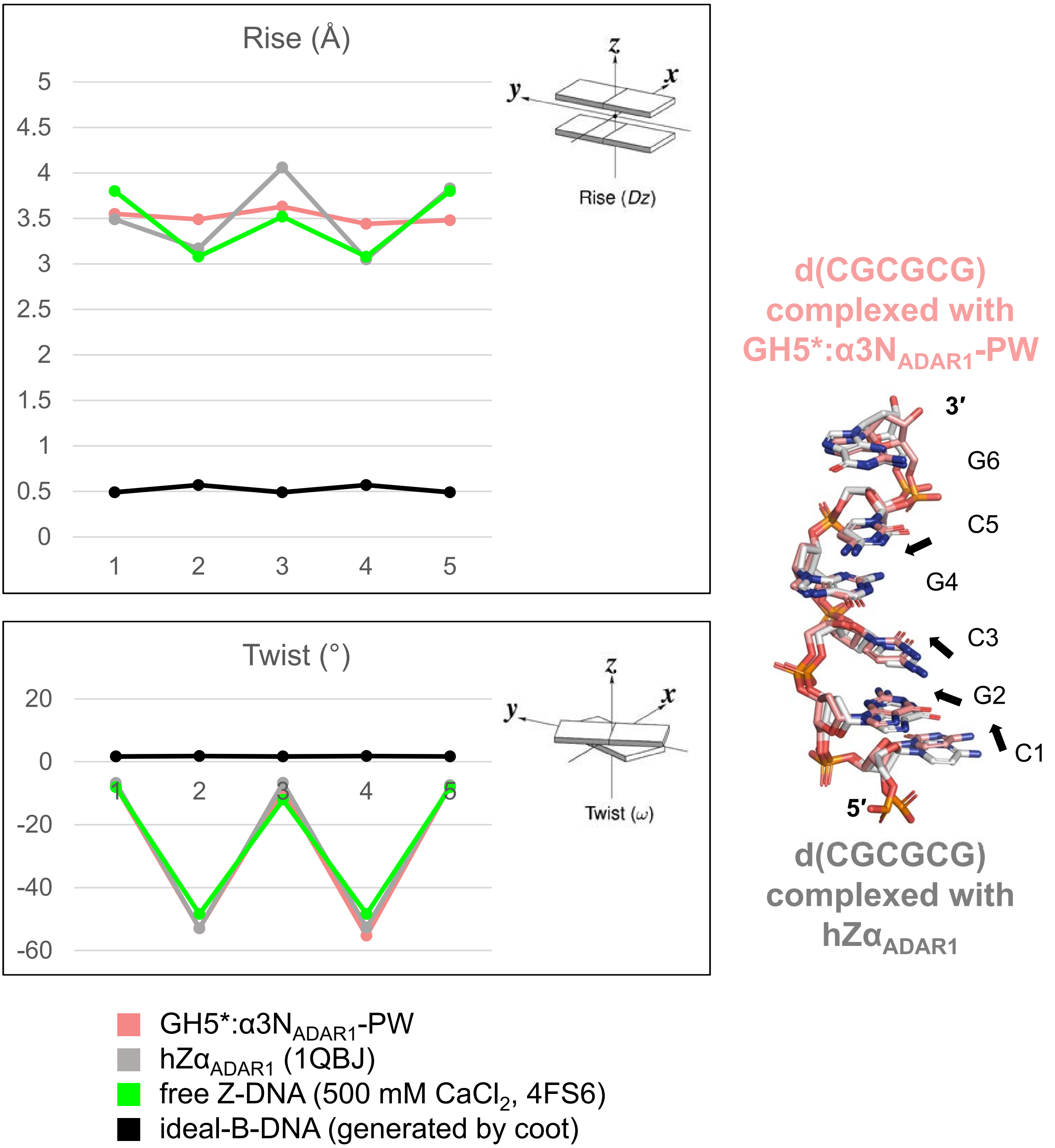

Figure S13. Structural analysis of the Z-DNA duplex from the GH5*:α3N$_{ADAR1}$-PW/ Z-DNA complex. Crystal structure of the Z-DNA duplex from the GH5*:α3N$_{ADAR1}$-PW/Z-DNA complex (salmon), hZα$_{ADAR1}$/Z-DNA complex (PDB ID 1QBJ, gray), free Z-DNA (PDB ID 3WBO, green), and ideal B-DNA generated by Coot (black) are analyzed by web 3DNA 2.0 (http://web.x3dna.org/analyze/). As in **Figure S9**, all four structures have the same sequence, CGCGCG. 'Rise' and 'Twist' clearly show that either free or protein-bound d(CGCGCG)$_2$ is different from ideal B-DNA, extended along the z-axis, and negatively twisted (left-handedness). Although GH5*:α3N$_{ADAR1}$-PW shows conserved interactions toward Z-DNA, the ‘Rise’ of the 2nd and 4th steps of GH5*:α3N$_{ADAR1}$-PW-bound Z-DNA is slightly bigger than that of hZα$_{ADAR1}$-bound Z-DNA, while the ‘Rise’ of the 3rd step shows the opposite pattern. This difference in ‘Rise’ pattern is caused by a slight shift toward G4 as shown by the black arrow.

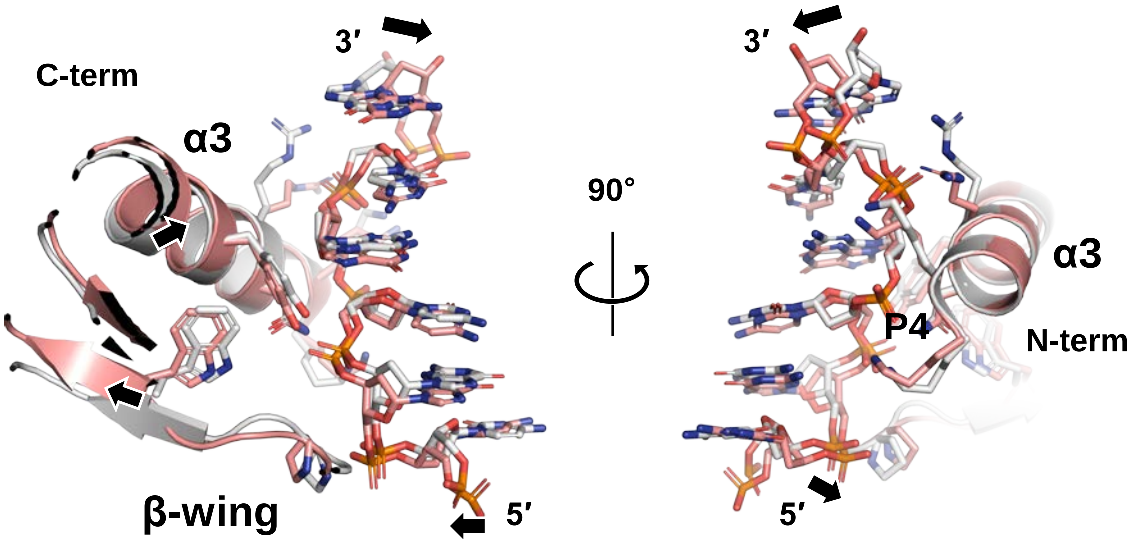

Figure S14. Structural alignment between the GH5*:α3N$_{ADAR1}$-PW/Z-DNA complex and the hZα$_{ADAR1}$/Z-DNA complex. Structure of GH5*:α3N$_{ADAR1}$-PW/Z-DNA complex (chain B and E, salmon) was aligned with the hZα$_{ADAR1}$/Z-DNA complex structure (PDB ID 1QBJ, gray). Alignment against the N-terminus of the α3 helix, which is shared by the two structures, revealed that the C-terminus of the α3 helix was moved about 1 Å, which results in movement of the β-wing and, finally, movement of nucleotides except for P4.

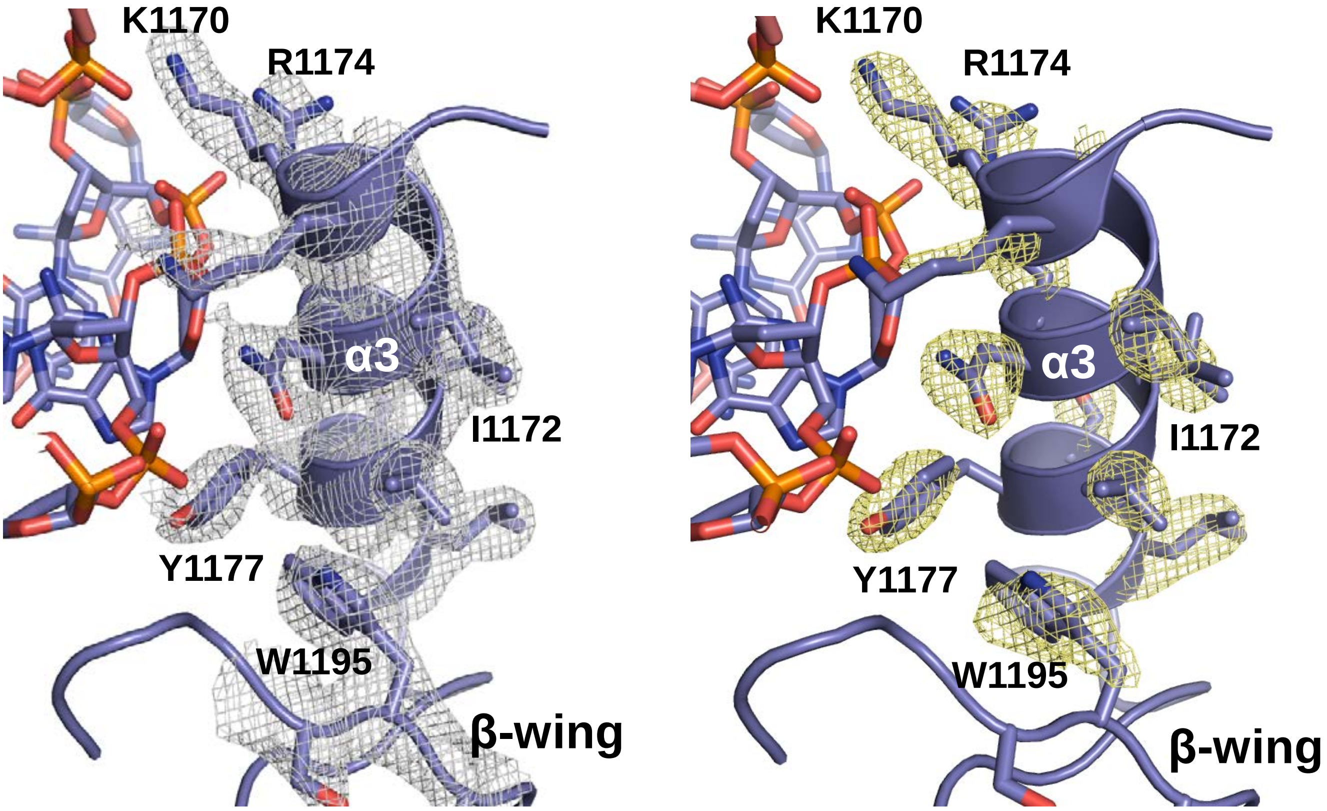

Figure S15. Composite omit map of the GH5*:α3N$_{ADAR1}$-PW/Z-DNA complex structure. Composite omit maps of 2mFo−DFc (left) and mFo-DFc (right) contoured at 1.0 σ and 3.0 σ levels, respectively, are shown for the α3 helix and β-wing of the GH5*:α3N$_{ADAR1}$-PW/Z-DNA complex. The electron density maps are shown as mesh (gray and yellow). Residues of α3 helix and β-wing participating in Z-DNA binding (R1174, Y1177, and W1195) are well defined.

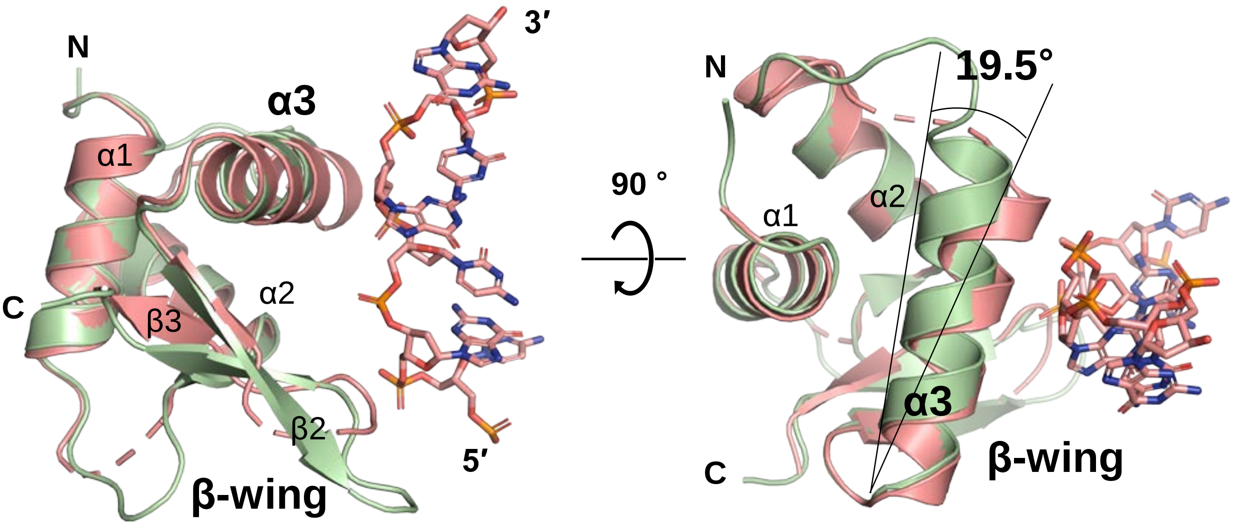

Figure S16. Structural alignment between GH5*:α3N$_{ADAR1}$-PW complexed with the Z conformation of [d(TCGCGCG)]$_2$ and free GH5. The structure of GH5*:α3N$_{ADAR1}$-PW (chain B, salmon) was aligned with the free GH5 structure (PDB ID 1HST, pale green). Overall alignment revealed that GH5*:α3N$_{ADAR1}$-PW maintained overall folding of GH5, but the α3 helix of GH5*:α3N$_{ADAR1}$-PW rotated toward Z-DNA by 19.5°. Dashed lines represent the unresolved region in the crystal structure.

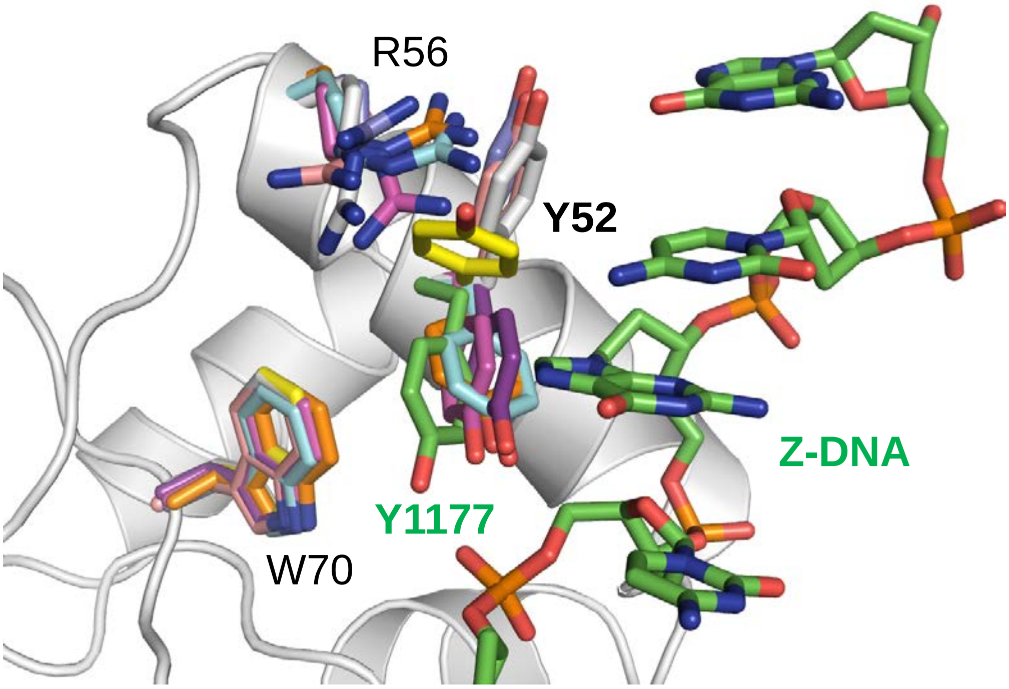

Figure S17. Conformational flexibility of vvZα$_{E3L}$ Y52 in a DNA-free state. The crystal structure of vvZα$_{E3L}$:α3$_{ADAR1}$ in complex with Z-DNA was aligned to the NMR structure of free vvZα$_{E3L}$ (PDB ID 1OYI). The Y1177 side chain and Z-DNA of the complex structure (chains A and D) are shown as green sticks. Among 8 representative models from 20 ensemble models of the vvZα$_{E3L}$ NMR structure, only one model is shown in a ribbon diagram (light gray) for simplicity. Side chains of Y52, R56, and W70 from 8 models are shown as sticks (different color for each model). Compared to nearby residues, Y52 shows multiple rotamer conformations in a DNA-free state.
